# A data-efficient and easy-to-use lip language interface based on wearable motion capture and speech movement reconstruction

**DOI:** 10.1126/sciadv.ado9576

**Published:** 2024-06-26

**Authors:** Shiqiang Liu, Terry Fawden, Rong Zhu, George G. Malliaras, Manohar Bance

**Affiliations:** ^1^ State Key Laboratory of Precision Measurement Technology and Instrument, Department of Precision Instrument, Tsinghua University, Beijing 100084, China.; ^2^ Department of Clinical Neurosciences, University of Cambridge, Cambridge CB23EB, UK.; ^3^ Electrical Engineering Division, Department of Engineering, University of Cambridge, Cambridge CB3 0FA, UK.

## Abstract

Lip language recognition urgently needs wearable and easy-to-use interfaces for interference-free and high-fidelity lip-reading acquisition and to develop accompanying data-efficient decoder-modeling methods. Existing solutions suffer from unreliable lip reading, are data hungry, and exhibit poor generalization. Here, we propose a wearable lip language decoding technology that enables interference-free and high-fidelity acquisition of lip movements and data-efficient recognition of fluent lip language based on wearable motion capture and continuous lip speech movement reconstruction. The method allows us to artificially generate any wanted continuous speech datasets from a very limited corpus of word samples from users. By using these artificial datasets to train the decoder, we achieve an average accuracy of 92.0% across individuals (*n* = 7) for actual continuous and fluent lip speech recognition for 93 English sentences, even observing no training burn on users because all training datasets are artificially generated. Our method greatly minimizes users’ training/learning load and presents a data-efficient and easy-to-use paradigm for lip language recognition.

## Teaser

A data-efficient method of fluent lip speech recognition is based on wearable motion capture and speech movement reconstruction.

## INTRODUCTION

Lip language decoding has recently attracted substantial interest due to its inherently high degree of privacy, resistance to acoustic noise, and the potential to enable verbal communication for patients with laryngectomies (*1*–*3*). It was initially considered as an alternative to voice-based speech recognition technology and showed promise in a broad range of areas including human-computer interfaces, health care, aerospace, and military. To date, a variety of lip language recognition systems have been reported, aiming at translating lip language into understandable contents such as texts or sounds automatically. However, limited by the interfacing and decoding technologies, these existing systems lag in being able to be practically used. There exist two bottleneck challenges. One is how to capture lip reading robustly and reliably with easy-to-use methods. The other is how to model lip language decoder data-efficiently to recognize fluent full sentences in real time, which is crucial for barrier-free real-life communication.

Existing lip language recognition approaches, often known as silent speech interfaces (SSIs) (*4*, *5*), primarily encompass vision-based methods (*6*, *7*), magnet-based methods (*8*–*10*), ultrasound-based methods (*11*, *12*), radar-based methods (*13*, *14*), and wearable sensor–based methods (*2*, *3*, *15*, *16*). The vision-based methods are susceptible to lighting conditions, camera angle, and occlusion issues. For example, cameras fail to work in the dark or when the user is wearing a face mask. In addition, they face challenges of heavy computing load and non-wearability and suffer from personal privacy issues. The magnet-based solutions are invasive and mainly designed for medical scenarios. The ultrasound-based solutions tend to be bulky and stationary. Moreover, the radar-based methods are also bulky and have restriction in spatial resolution. Wearable sensor–based methods, which have been broadly investigated for motion monitoring and human-computer interaction, are becoming more and more promising in this field. Existing wearable methods of lip language recognition usually adopt small and light-weight sensors, including surface electromyography (sEMG) sensors (*15*, *17*–*20*), stretchable strain sensors (*1*, *3*), triboelectric sensors (*2*), and inertial sensors (accelerometers and gyroscopes) (*16*, *21*), aiming at comfortable daily use (*4*, *5*).

However, sEMG has limitations of poor long-term stability, low signal-to-noise ratio, and susceptibility to muscle fatigue (*22*, *23*). Stretchable strain sensors have been reported to have better performance than sEMG in lip language recognition, such as a SSI using crystalline silicon–based strain gauges in (*3*) and an artificial throat using graphene strain sensors in (*1*). However, the strain sensors generally have poor stability and degrade obviously after stretching (*3*) or bending (*1*) cycles. An interface based on triboelectric sensors is developed for lip language decoding while a face mask is worn (*2*), whereas the sensors suffer from unsatisfactory signal-to-noise ratio and poor repeatability and only respond to dynamic motion with a limited bandwidth (*2*). Accelerometers have also been adopted for SSI by using the sensors’ raw outputs, which have outperformed sEMG obviously (*16*). However, accelerometers’ raw outputs are susceptible to the orientation versus gravity and the movements of the head and body and therefore suffer from unwanted artifacts (*24*, *25*). Therefore, it is challenging to capture lip reading robustly and reliably with existing wearable solutions. Because of these inherent constraints, almost all existing wearable solutions focus only on simple demonstration of word/command-level recognition. As mentioned above, another challenge is data-efficiently modeling of lip language decoder to recognize fluent full sentences in real time for barrier-free real-life communication. Up to now, deep learning method has provided the most popular paradigm for decoding and recognition (*26*, *27*). A large amount of annotated speech datasets (e.g., with sentence/word/phoneme element labeled) from sentence speech are required for decoders’ modeling (*28*, *29*) in these continuous expression recognition scenarios. This problem is still more challenging for lip language applications, where the captured speech information is especially specific to the individual (*5*), meaning that large volumes of data need to be collected from every user and huge training load takes place. Therefore, there is a great need to investigate wearable and easy-to-use interfaces for interference-free and high-fidelity lip-reading acquisition and to develop accompanying data-efficient decoder-modeling methods. This would greatly facilitate the practical application of lip language recognition but has not been sufficiently studied and developed yet.

**Fig. 1.**
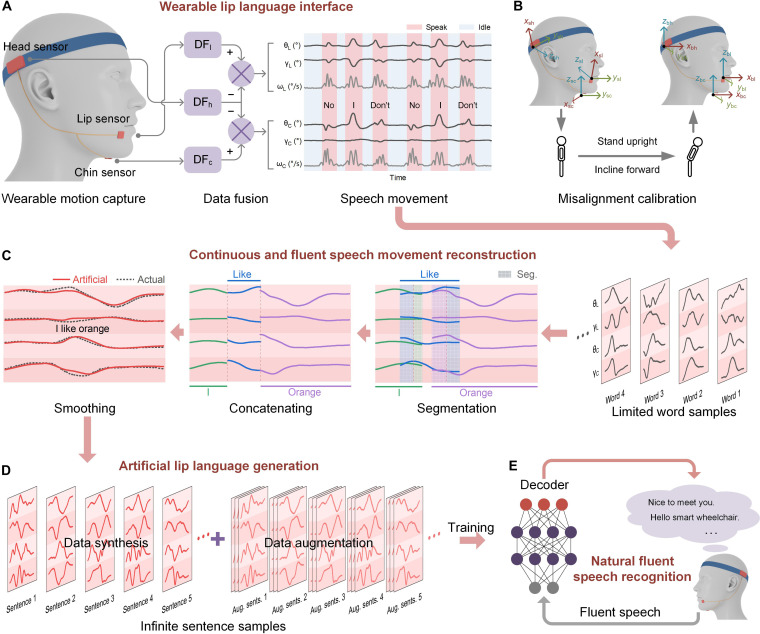
A data-efficient and easy-to-use method for natural fluent lip language recognition based on wearable motion capture and speech movement reconstruction. (**A**) The wearable motion capture system captures the relative movement information (relative angles) of the lip and chin versus the head. DF means data fusion. The subscript refers to the first letter of lip, chin, or head. (**B**) Misalignment calibration for eliminating the misalignment errors of the sensors. (**C**) Artificial generation of natural fluent lip speech data from independent words by using the proposed strategy for speech movement reconstruction. (**D**) Infinite continuous and fluent lip speech data can be generated from a limited word corpus based on the strategy of artificial data synthesis and data augmentation. (**E**) By using the artificially generated corpus of fluent speech data, a decoder is modeled and actual natural fluent lip speech recognition is realized.

Here, we put forward a wearable and easy-to-use lip language recognition technology that enables interference-free and high-fidelity acquisition of lip movements and data-efficient modeling of lip speech decoders based on our proposed wearable motion capture system and our speech movement reconstruction strategy (Fig. 1). Relying on a limited training word corpus from users, it works robustly and data-efficiently for real-time recognition of both independent words and continuous fluent lip speech and effectively reduces the training burden on users.

We design a wearable motion capture system to capture the relative movements of lip and chin versus head (Fig. 1A), including the relative attitude angles of pitch and roll and the relative three-axis angular velocities by using a custom data fusion algorithm (*30*–*33*). Besides, the misalignments are eliminated through an easy-to-implement calibration (Fig. 1B). Compared to existing research using the raw outputs of accelerometers and gyroscopes, our method is able to provide interference-free and high-fidelity acquisition of lip movements with no misalignment errors and artifacts arising from head/body motion. Therefore, we realize a robust and reliable interface for lip language decoding based on wearable motion capture. The effectiveness of our lip language interface is first validated for individual word recognition, with a custom decoder based on a deep learning network. An average accuracy of 97.4% is observed across individuals (*n* = 8) for 93 commonly used English words, with a training dataset ratio of only 30% per word (20 samples in total; training/test, 30%/70%) for decoder modeling. This greatly reduces the training load on users compared to existing methods, where at least 70% of datasets are required to participate in decoder training to achieve a satisfactory accuracy.

In addition, we propose a strategy of speech movement reconstruction to artificially synthesize natural fluent lip speech data from independent words into continuous and fluent sentences by concatenating waveforms of the contained words with proper segmentation (Fig. 1C). A custom Bayesian optimization method is developed to estimate the general segmentation parameters for natural fluent speech. The segmentation parameters exhibit a similar distribution between individuals, allowing our method to generalize well. We artificially synthesize samples of 93 English sentences using general segmentation parameters and then use them to train a custom temporal convolutional network (TCN)–based deep learning model for continuous fluent lip speech recognition (Fig. 1, D and E). An average accuracy of 92.0% across individuals (*n* = 7) is achieved when recognizing the 93 sentences while actually spoken in a natural and fluent way. This causes no training load on users because all training samples are artificially synthesized. Therefore, our method greatly minimizes users’ training/learning load, gives a data-efficient paradigm for natural fluent lip language recognition, and provides a promising strategy for semi-supervised or unsupervised learning.

## RESULTS

### Interference-free and high-fidelity acquisition of lip speech

We design a wearable motion capture system to capture the relative movements of lip and chin versus head for lip language recognition (Fig. 1A and fig. S1), including the relative attitude angles of pitch and roll and the relative three-axis angular velocities. It works online in real time for lip language recognition, including sensor sampling and data fusion, as well as speech detection and recognition. An easy-to-implement online calibration is adopted to eliminate misalignment errors between sensors and human body segments (jaw, lip, and head). The motion artifacts exist in the lip and chin inertial-measurement-unit (IMU) sensors, caused by the movements of the head and body, are measured by the IMU sensor attached on the head, and are eliminated by subtracting the head movements from those of the lip and chin sensors. A custom data fusion method based on Kalman filter is developed for the relative movement information extraction and the suppression of vibration interference and cumulative drift errors at the same time. Details about misalignment elimination and data fusion can be found in Materials and Methods.

**Fig. 2.**
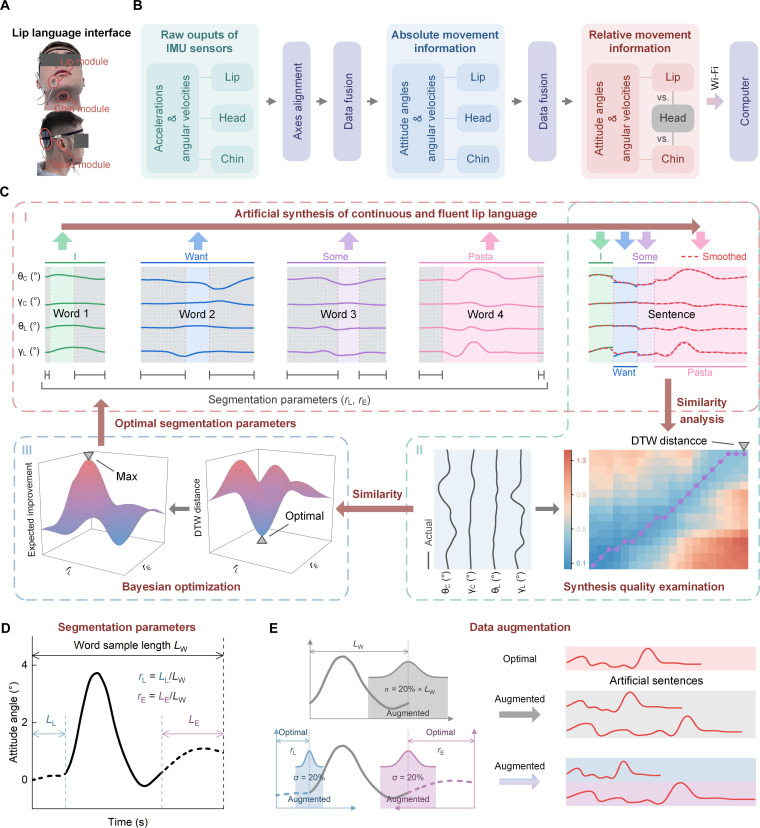
The operating principle of the wearable lip language interface and the artificial generation strategy for producing continuous fluent lip language data. (**A**) Photograph and (**B**) the schematic diagram of the wearable lip language interface. (**C**) Artificial generation strategy of continuous fluent lip language data, based on speech movement reconstruction (Sub. 2 for example). (**D**) Definition of word segmentation parameters during speech movement reconstruction. (**E**) The principle of data augmentation for artificial fluent lip language generation to improve the generalization.

Our wearable lip language recognition system adopted a headband design (Fig. 1A and fig. S1) to allow it to be worn comfortably. It consists of one headband, one head module with a built-in IMU sensor attached to headband (the head sensor), and two tiny IMU sensor modules attached to the skin of the lip (the lip sensor) and chin (the chin sensor), respectively. Here, the head sensor captures the head movement of the user. The lip sensor captures the movement of the mouth muscles. The chin sensor, attached on the middle of the skin below the chin, is able to capture the combined movement that incorporates elements of movements of the mandible, tongue, and throat. The misalignment elimination is conducted online when the user wear the lip language recognition system by virtually aligning the local sensor coordinate systems to the body segment coordinate system through an easy-to-implement two-phase calibration procedure (Fig. 1B) (*33*). The data fusion is carried out on the basis of a custom Kalman filtering method (fig. S2). Kalman filters have been shown to effectively suppress drift and interference errors in the IMU outputs, including shock and vibration interference in the accelerometer and cumulative drift errors in the gyroscope (*30*). Last, we realize interference-free and high-fidelity acquisition of lip speech. Figure 2B shows this in a schematic diagram.

We validate the performance of our system through experiments. First, the misalignment elimination is evaluated using a high-precision tri-axis turntable. The chin and lip sensors as well as the head sensor are mounted on different frames of the turntable. The setup is used to simulate the chin and lip movements versus head of a user. In the experiment, the misalignment calibration is conducted by rotating the sensors following the calibration procedure. Then, the chin and lip sensors are rotated versus the head sensor to generate the relative movements. The turntable provides the true angles of these relative rotations. Three independent tests with different misalignments are conducted to simulate the repeated uses of the user. The measurement results of the chin sensor and the lip sensor versus the head sensor are shown in figs. S3 and S4, respectively. The results show that the measurement errors of relative movements (chin/lip versus head) by using our system are greatly reduced from about 39.30° to less than 0.35° when the chin and lip move from −60° to 60° around the *x* and *y* axes of the head coordinate system, respectively. The misalignment elimination ensures the consistent performance for the repeated uses. Details of experimental setup are described in Materials and Methods.

Then, the effectiveness of interference resistance is validated. For this, our wearable lip language recognition system is fixed on the surface of an aluminum cube, which is used to imitate a human head. The cube is rotated around two different axes successively to generate vibrations in attitude angles. The experiment setup was selected to simulate the head movement of a person who is not speaking, where there are no relative movements of the lip and chin versus head. Misalignment elimination is conducted first. In addition, the system outputs lip speech information with the proposed data fusion method (relative attitude angles of the lip and chin versus head). The results are shown in fig. S5, where the relative attitude angles (θ_L_, θ_C_, γ_L_ and γ_C_) represent the system errors caused by head movement interference due to no speech activity. It can be seen that the absolute values of θ_L_ and θ_C_ stay below 0.23°, while the absolute pitch angle of the head $\theta_{H}^{\text{abs}}$ varied from −25.60° to 19.33°. In addition, the absolute values of γ_L_ and γ_C_ stay below 0.32°, while the absolute roll angle of head $\gamma_{H}^{\text{abs}}$ varied from −18.28° to 26.80°. Therefore, our lip speech interface is almost entirely free from interference and can be used for high-fidelity motion capture of lip speech.

### Artificial synthesis of natural fluent lip speech

Natural conversation is underpinned by continuous and fluent speech using sentences in daily life. Current supervised learning–based methods for speech recognition require too much training data to be practical for unrestricted communication, especially for lip language, due to its highly individual specificity. To address this problem, we propose a strategy for speech movement reconstruction to artificially synthesize continuous fluent lip speech data from independent words based on our lip language interface (Fig. 2C). We use the relative attitude angles of the lip and chin to represent features of lip language. We reconstruct the movement waveforms of the lip and chin for naturally and fluently spoken sentences by concatenating the related words’ waveforms with proper segmentation at the lead and end (Fig. 2C). The segmentation parameters of a word are defined as the ratios of lead/end length to be segmented divided by the word waveform length (Fig. 2D). The waveforms of a randomly selected full sentence and the corresponding individual words from Sub. 2 are used for example here (Fig. 2, C to E). A method based on Bayesian optimization (*34*–*36*) is developed to estimate the segmentation parameters while reconstructing full-sentence waveforms from individual words (Fig. 2C). The objective function of Bayesian optimization is defined as the similarity between the synthesized and actual sentences and is scaled by custom dynamic-time-warping (DTW) distance that has been selected to evaluate the similarity between two sequence data having potential common features with different length (*37*–*40*). Bayesian optimization gives the optimal segmentation parameters by minimizing the defined DTW distance. Thus, the optimal segmentation parameters of words in different sentences are estimated and lastly used for continuous fluent lip speech data generation.

We find that the segmentation parameters tend to exhibit similar distributions between individuals during natural fluent speech, which allows us to transfer parameters from one person to another. Results are given in the “Continuous and fluent lip language recognition” section. Considering the potential fluctuations of speaking speed and rhythm in the same person and between persons, data augmentation is conducted during continuous fluent lip speech data generation to enhance generalizability, using the optimal segmentation parameters and the Monte Carlo method (Fig. 2E). This allows us to artificially generate “infinite” datasets of any continuous and fluent lip speech as needed, depending only on a limited word corpus. Hence, it minimizes the training/learning burden on lip language users.

### Independent word recognition

**Fig. 3.**
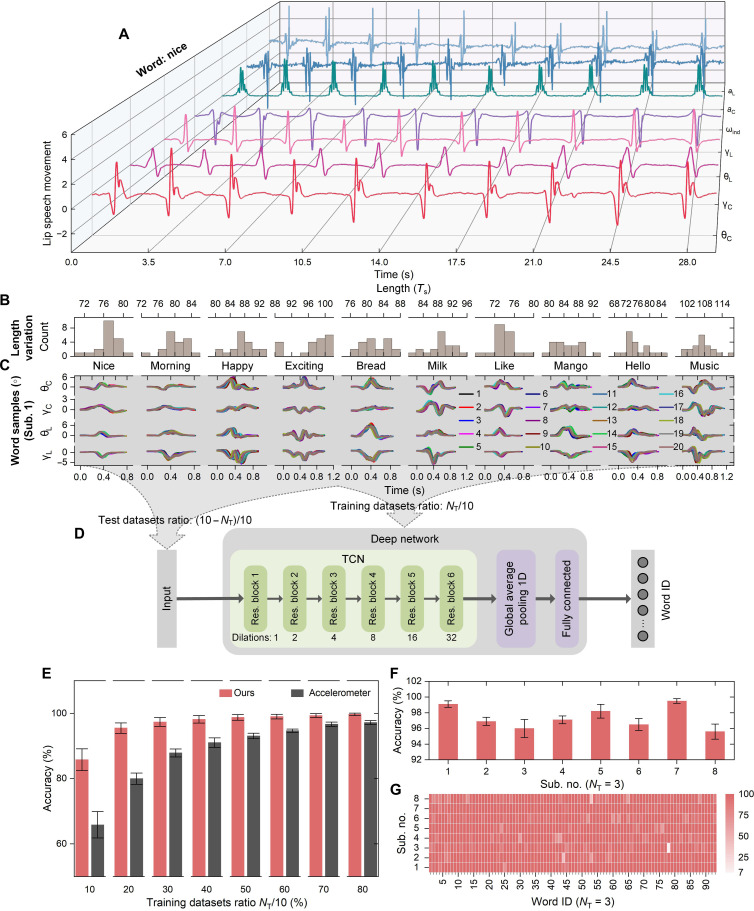
The results obtained using individual-word–based speech recognition. (**A**) Movement information outputted by our lip language interface during word speech (Sub. 1), the spoken word here is “nice” that is randomly selected from the results. (**B**) The sample length distribution of 10 randomly selected words and (**C**) the waveforms of these word samples (Sub. 1). Twenty samples of each word are used for statistical analysis. (**D**) A TCN-based neural network is used for individual-word–based speech recognition. (**E**) Results of cross validation with the training dataset ratio varying from 10 to 80%. The red and gray columns represent the results of our method (Ours) and from just using the raw accelerometer outputs (Accelerometer), respectively. (**F**) Word recognition performance across individuals and (**G**) accuracy by word of each individual while using six samples per word (training dataset ratio of 30%) for network training. Sub., participant.

We first carried out experiments on word speech recognition to validate the effectiveness of our lip language interface. This involved eight participants including four native English speakers and four non-native yet proficient English speakers. Ninety-three English words that are commonly used in social and emotional contexts, for expression of basic needs and in human-robot interaction (HRI) scenarios, were selected (table S1). The participants repeat the words one by one (20 times per word) and keep their mouths still in-between in a relaxed, self-selected way. The captured relative attitude angles (θ_L_, γ_L_, θ_C_, and γ_C_), angular velocity indicator (ω_ind_), and some of raw accelerometer outputs (*a*_L_ and *a*_C_) of the lip and chin during repeated speech of one of the words are shown in Fig. 3A. ω_ind_, *a*_L_, and *a*_C_ have been rescaled to display here (details in fig. S6). The waveforms and corresponding length variations of 10 randomly selected words (20 repetitions per word, Sub. 1) are shown in Fig. 3 (B and C).

Our results clearly indicate that the relative attitude angles of the lip and chin exhibit great consistency in features of the same word and obvious differences between words. This fundamentally explains why our wearable motion capture system allows for robust and reliable lip language decoding. We then develop a TCN-based deep learning neural network (Fig. 3D) for decoder modeling and word speech recognition. TCN neural networks are an effective tool for time sequence feature extraction and classification, and we therefore adopt one here for lip language decoding (*41*). Network hyperparameter optimization is shown in fig. S7. Tenfold cross-validation tests with randomly mixed datasets were performed, where the 20 datasets, comprising 93 words each, were randomly divided into 10 folds. One to eight folds (*N*_T_) were increasingly selected for network training, whereas the rest are used to test the network (Fig. 3, C and D). The average recognition accuracy of each cross-validation setup was used for the final evaluation. The red columns in Fig. 3E present the average accuracy of word recognition across individuals (*n* = 8, including four native speakers and four non-native speakers) versus the training dataset ratio of each word. The accuracy increases with training sample size and reaches 97.4% when using three folds (six samples or 30% per word) for training. Our method allows for accurate lip language recognition using only 30% of the datasets per word for decoder modeling and, therefore, greatly minimizes the training load on users.

Our method greatly outperforms the method of simply using the raw accelerometer outputs on both accuracy and data dependency (Fig. 3E). Figure 3F gives the recognition performance among different individuals, and Fig. 3G shows the recognition accuracy achieved using word by word for each individual in detail, with training dataset ratio 30%. These results validate the reliability and robustness of the proposed lip language recognition interface. Additional details of the experimental protocol and data analysis methodology are given in Materials and Methods.

### Continuous and fluent lip language recognition

**Fig. 4.**
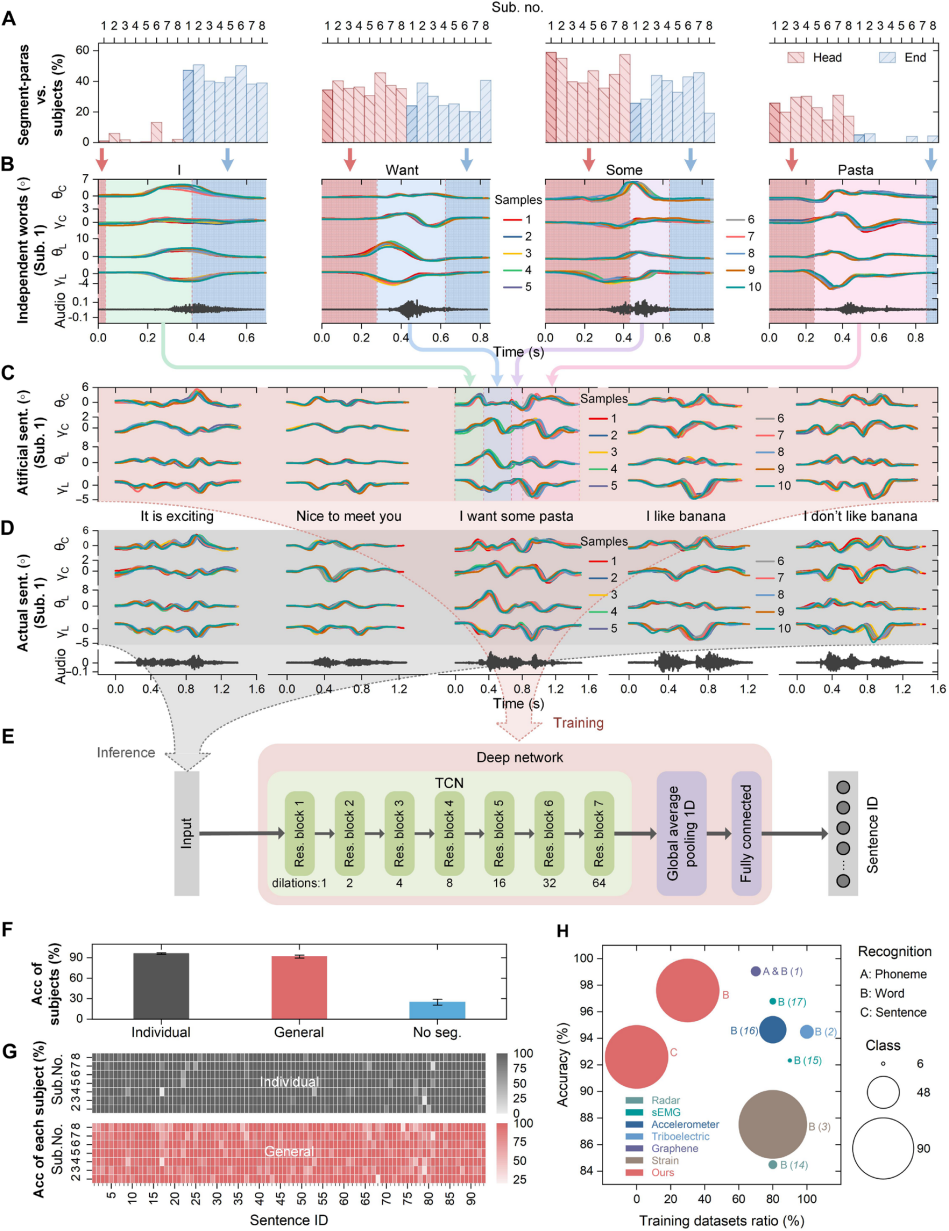
The results of artificial data generation of continuous and fluent lip language and full-sentence–based speech recognition. (**A**) Optimal segmentation parameter distribution between individuals. (**B**) Ten samples of the words “I,” “want,” “some,” and “pasta” (Sub. 1). (**C**) Ten artificially generated samples of the five different sentences including “I want some pasta” (Sub. 1). (B) and (C) The application of lip language data synthesis to produce fluent sentences from individual words. (**D**) Ten actual samples of the five sentences, during natural fluent speech (Sub. 1). (**E**) A TCN-based neural network, developed for sentence speech recognition. (**F**) Average recognition accuracies for the 93 sentences across all individuals using only artificial training datasets synthesized using individual, general, and no seg. parameters. (**G**) Recognition results by sentence from Sub. 2 to Sub. 8, using individual (gray) and general (red) synthesis parameters, respectively. (**H**) Comparison to state-of-the-art research on lip language recognition.

Experiments were also conducted to evaluate the effectiveness of our speech movement reconstruction strategy to generate continuous and fluent lip speech data and the method of data-efficient continuous and fluent lip language recognition. The same eight participants participated in these trials. Ninety-three English sentences were selected (table S2), composed of words from the above 93 words. The full-sentence speech trials followed a similar protocol to that of the individual-word–based speech, with 10 samples per sentence collected. To validate our artificial speech data synthesis method, all the word samples acquired in the individual-word–based speech experiments were used, and 20 artificial samples were synthesized for each sentence by concatenating 20 samples of every word contained in the sentence after having been suitably segmented. On the basis of the Bayesian optimization method, the segmentation parameters of words for sentence synthesis were first recognized. The sentence “I want some pasta” is randomly selected for demonstration, and the optimal segmentation parameters across individuals for the four words that it contains are illustrated in Fig. 4A, where less than 26.5% variation can be observed between individuals due to the differences in speech speed and mannerisms, despite this being a mixture of native and non-native English speakers. The results of natural fluent sentence synthesis from one participant (Sub. 1) are shown in Fig. 4 (B to D), where Fig. 4B illustrates the synthesis process of one sentence, Fig. 4C presents synthesized samples of five randomly selected sentences (10 samples per sentence are presented), and Fig. 4D gives the actual lip speech samples of those five sentences (10 samples per sentence). Similarity can be observed between the artificial sentence waveforms and the actual ones. It is noted that, the voice is just recorded for reference. The lip movements while speaking are usually much longer in time than the accompanying voice because the preparation and ending movements exist.

We then used a TCN-based deep network (Fig. 4E) (*41*) to model the decoder for continuous and fluent lip language recognition, where only artificially generated sentence samples were used for model training and the actual sentence samples were for testing. Artificial datasets for all 93 sentences were generated and augmented as follows: First, 20 artificial samples were generated for each sentence using the optimal segmentation parameters. Then, another 2000 samples were generated for each sentence according to our proposed data augmentation method, with a SD of 20% for the Monte Carlo process. Thus, 2020 artificially generated and augmented samples were produced for each sentence and used for subsequent network training. Network hyperparameter optimization can be found in fig. S8.

Three scenarios were designed to further evaluate our method’s effectiveness and independence from test participants: scenario 1, where all participants use their own optimal segmentation parameters to generate the artificial sentence datasets (Individual in Fig. 4F); scenario 2, where all participants share the optimal segmentation parameters from one given participant (Sub. 1, non-native yet proficient speaker) to generate the artificial sentence datasets (General in Fig. 4F; table S3); and scenario 3, where all participants artificially generate sentence data directly without segmenting the word samples (No seg. in Fig. 4F). The average recognition accuracy for the 93 sentences across all participants (*n* = 7, including four native speakers and three non-native yet proficient speakers) is 96.4% with individual parameters (figs. S9 to S15), 92.0% with general parameters (figs. S16 to S22), and 25.1% with zero parameters, respectively. The recognition accuracies by sentence of each individual (Sub. 2 to Sub. 8) using individual and general parameters are shown in Fig. 4G. It shows that our method works robustly and generally among individuals although specific, personalized differences exist. In other words, our strategy of artificial sentence generation reveals the common and fundamental mapping mechanism from independent basic elements, e.g., individual words, to continuous, complex speech content, e.g., full sentences during natural fluent speech, from the perspective of the mechanical movements that produce speech. Further, the proposed data augmentation competently covers the individual differences in mannerisms during natural speech. It means that we only need to calibrate the segmentation parameters for natural speech synthesis from a small subset of users and then can transfer them to a large population. Therefore, we realize a data-efficient method of continuous and fluent lip language recognition based on our lip language interface and artificial lip language data generation strategy. Movies S1 and S2 show that our method works robustly for recognizing sentence-level speech (Sub. 1) while speaking under different speed (normal and slow) and while speaking both vocally and silently.

Comparison to state-of-the-art research on lip language recognition is summarized in Fig. 4H, including those based on radar, sEMG, accelerometer, triboelectric sensor, graphene mixed-modality sensor, strain gauge, and our method. Most of them only worked for a very limited corpus of words, except Kim *et al*. (*3*) who have extended the word corpus to 100. Researchers in (*1*) tried to demonstrate their feasibility for sentence recognition but only presented six very short sentences from one participant. What is more, all existing research has required a training dataset ratio of at least 70% (tens or even hundreds of training samples per word) to achieve a satisfactory recognition performance. It is apparent that our method realizes data-efficient recognition of a much larger corpus of words and sentences while only using limited training datasets of words. We achieve accurate recognition of continuous and fluent lip speech with even no training load on users.

## DISCUSSION

In this work, we conceptually propose a wearable solution for lip language decoding, based on our wearable motion capture system and artificial lip language data generation strategy. It provides a lip language interface to read lip movement information with high resistance to interference and high fidelity. This solution enables the efficient decoding and recognition of continuous and fluent lip speech by means of the proposed artificial data generation strategy. The effectiveness of our method was validated by experiments. For individual word recognition, we achieve an average accuracy 97.4% across individuals (*n* = 8) for 93 commonly used English words while using only six samples (training dataset ratio of 30%) per word for decoder model training. Compared to existing lip language recognition methods, our method greatly reduces the training load on users. Furthermore, we artificially synthesized natural fluent sentence data from independent word samples and used them to train decoders for continuous lip speech recognition. An average accuracy of 92.0% across individuals (*n* = 7) was observed while recognizing 93 sentences when naturally and fluently spoken, causing no training load on users because all training datasets were artificially generated. Our artificial speech data generation strategy exhibits excellent generalizability among individuals. This allows more flexibility for research on lip language recognition, particularly early-stage modeling, by generating infinite continuous and fluent speech data using a very limited word corpus. It greatly minimizes users’ training/learning burden, creates a data-efficient paradigm for lip language recognition, and provides a promising strategy for semi-supervised or unsupervised learning.

## MATERIALS AND METHODS

### Design of the wearable lip language recognition system

As shown in Fig. 1A and fig. S1, our lip language recognition system adopts a headband design that allows it to be worn comfortably for long durations. It consists of one headband; one head module (edge computing unit, Arduino Nano 33 BLE, Arduino S.r.l.) with a built-in IMU sensor (LSM9DS1, STMicroelectronics) attached to headband (the head sensor); two tiny IMU sensor modules (LSM9DS1, STMicroelectronics) attached to the skin of the lip (the lip sensor) and chin (the chin sensor), respectively; and one wireless transmitter (Arduino Nano 33 IoT, Arduino S.r.l.). Here, the head sensor captures the head movement of the user. The lip sensor captures the movement of the mouth muscles. The chin sensor, attached on the middle of the skin below the chin, is able to capture the combined movement that incorporates elements of movements of the mandible, tongue, and throat. The head sensor is attached on the lateral posterior aspect of the parietal bone by using an elastic head band. The lip and chin sensors are attached on the skin of the lip and the chin, respectively, using medical tape. The setup ensures that the sensors can keep their position and attitude on the skin during the chronic usage. Both the accelerometers and gyroscopes of the three IMUs are used; therefore, three-axis accelerations and three-axis angular velocities are reported for each. The measurement ranges of the accelerometers and gyroscopes are set as −2 to +2 g and −200°/s to +200°/s, respectively, where g refers to the acceleration due to gravity. The two tiny IMUs are connected to the edge computing unit via an inter-integrated circuit bus. The edge computing unit samples the three IMUs synchronously at 100 Hz, calculates their absolute attitude angles of pitch and roll based on our custom Kalman filters (fig. S2) (*30*), and lastly gives the relative attitude angles and relative angular velocities of the lip and chin versus the head in real time based on our custom data fusion method. The schematic diagram is shown in Fig. 2B. The edge computing unit communicates with the Wi-Fi module via its universal asynchronous receiver/transmitter (UART) port and wirelessly transmits the relative movement information of the lip and chin to a host computer in real time. The wearable system is powered by a lithium-ion battery. The host computer (CPU: i5-8279U, Intel) runs our custom software using Python (version 3.9) and is responsible for real-time speech detection, sample extraction, lip language recognition, results display, and data storage. The entire response time of our system including the operation on Python TensorFlow and Arduino is only about 85.7 ms, including 3.7 ms for sensors’ sampling and edge computing on Arduino, 1.6 ms for UART transmission, 3.8 ms for Wi-Fi transmission, only 10 μs of Python software response, and 76.6 ms for neural network inference calculation (CPU i5-8279U, Intel).

### Misalignment elimination

In this section, we present the method to align the local sensor coordinate systems to the respective body segment coordinate systems and to eliminate the misalignment errors. Note that all the IMUs have been calibrated in advance to correct for the measurement errors in the accelerometers and gyroscopes.

Here, the IMU sensors’ coordinate systems *x*_si_-*y*_si_-*z*_si_ are aligned to the body coordinate systems *x*_bi_-*y*_bi_-*z*_bi_ of the head, lip, and chin, respectively (Fig. 1B), where the subscript i refers to the corresponding first letters of “head,” “lip,” and “chin.” The coordinate systems *x*_bi_-*y*_bi_-*z*_bi_ are not anatomically derived, rather they are defined during the alignment of the coordinate systems. They can be visualized simply as follows: While the user stands upright and keeps their mouth closed, *z*_bi_ points up vertically, *x*_bi_ points forward in the sagittal plane, and *y*_bi_ points left to form a right-handed coordinate system with *x*_bi_ and *z*_bi_. A calibration procedure consisting of two phases is used to estimate the misalignment angles between the sensor and head coordinate systems (*31*–*33*). In the first phase, the user stands upright; in the second phase, the user leans forward, causing an incline of the trunk and head. During each phase, the user keeps their posture for 5 s. To avoid the influence of jaw and lip movements on the alignment, the user keeps the mouth closed and the teeth clenched during the entire procedure of the misalignment elimination. After calibration, the misalignment errors between sensors and the body are eliminated. From this point on, only the body coordinate systems *x*_bi_-*y*_bi_-*z*_bi_ are considered.

Validation experiments are conducted by using a high-precision tri-axis turntable (SGT-3, Beijing Precision Engineering Institute for Aircraft Industry). The head sensor is fixed on the middle frame of the tri-axis turntable, and the chin and lip sensors are fixed on another single-axis turntable mounted on the inner frame of the tri-axis turntable, so that the chin and lip sensors can rotate along two orthogonal axes versus the head sensor. In the experiment, the sensors are rotated synchronously around the middle axis for the misalignment calibration following the calibration procedure. Then, the chin and lip sensors are rotated versus the head sensor along the inner axis (*x* axis in figs. S3 and S4) and the middle axis (*y* axis in figs. S3 and S4) to generate relative movements. Relative movement from −60° to 60° along each axis is generated with a step of 15°, covering the movement range of the mouth. Three independent tests with different misalignments are conducted to simulate the repeated uses of the user. As shown in figs. S3 and S4, the maximum measurement errors of the relative movements by using our system in test 1, test 2, and test 3 reach 8.21°, 39.30°, and 29.29°, respectively, before misalignment elimination, which are caused by the different misalignments. However, the errors of our system in test 1, test 2, and test 3 are only less than 0.33°, 0.35°, and 0.34°, respectively, after misalignment elimination.

### Data fusion

In this section, we present the data fusion method applied to calculate the relative movement information of the lip and chin versus the head. The data fusion can be divided into two steps: (i) calculation of the absolute attitude angles of the head, lip, and chin from the IMUs’ raw measurements using Kalman filtering; and (ii) calculation of the relative movements of the lip and chin versus the head.

First, a custom Kalman filter is applied to calculate the absolute attitude angles of the head, lip, and chin from the raw outputs of the accelerometers and gyroscopes. The absolute attitude refers to the pitch and roll angles of the frame *x*_bi_-*y*_bi_-*z*_bi_ versus the global coordinate frame *x*_n_-*y*_n_-*z*_n_. During the following analysis, the head is arbitrarily chosen for means of demonstration, with the same methods applying for the lip and chin locations. *x*_bi_-*y*_bi_-*z*_bi_ is simplified as *x*_b_-*y*_b_-*z*_b_, and *x*_n_-*y*_n_-*z*_n_ is defined as the north-west-up frame. Further, *x*_b_-*y*_b_-*z*_b_ is assumed initially to be coincident with *x*_n_-*y*_n_-*z*_n_ to simplify the analysis.

Kalman filters have been shown to effectively suppress the drift and interference errors in the IMU outputs, including shock and vibration interference in the accelerometer and cumulative drift errors in the gyroscope (*30*). Notably, IMU readings taken from head movements are much less prone to noise than those taken from other human body segments due to the inherent stabilization mechanism of the head, alongside the vibration damping caused by soft tissues and bone structures. Therefore, accurate attitude angles of the head can be calculated using Kalman filters. In our application, the linear acceleration **a**_b_ and gravitational acceleration **g**_b_ in *x*_b_-*y*_b_-*z*_b_ are selected as the state variables for Kalman filtering, as shown in Eq. 1

$$X=\left[\begin{array}{c} a_{b}\\ g_{b} \end{array}\right]$$ (1)

To describe the temporal behavior of a segment’s linear acceleration, we use a stochastic method based on the Gauss-Markov (GM) model, as shown in Eq. 2. Here, the process error of linear acceleration **w**_a_ is assumed to be white Gaussian noise with a bias of zero and the SD σ_a_. η is constant for the GM model

$${\dot{a}}_{b}=\eta a_{b}+w_{a}$$ (2)

The acceleration due to gravity, **g**_b_, has constant value but variable orientation in *x*_b_-*y*_b_-*z*_b_ with the head’s rotation. It has been shown that the variation of **g**_b_ follows the relationship displayed in Eq. 3 with angular velocity **ω**_b_

$${\dot{g}}_{b}=-\left[\omega_{b}\times \right]g_{b}$$ (3)

$$\omega_{b}=\left[\begin{array}{c} \omega_{xb}\\ \omega_{yb}\\ \omega_{zb} \end{array}\right]$$ (4)

$$\left[\omega_{b}\times \right]=\left[\begin{array}{ccc} 0 & -\omega_{zb} & \omega_{yb}\\ \omega_{zb} & 0 & -\omega_{xb}\\ -\omega_{yb} & \omega_{xb} & 0 \end{array}\right]$$ (5)

With the simplification that the angular velocity remains constant during each sampling period *T*_s_, the discrete-time state equation for the Kalman filter is then established from Eqs. 2 and 3, as shown in Eqs. 6 and 7. Here, *k* refers to the *k*th sampling period, **W**^*k*^ is the process error whose covariance matrix **Q**^*k*^ can be derived according to Eq. 8, and σ_g_ is the SD of the gyroscope outputs

$$X^{k+1}=\Phi^{k}X^{k}+W^{k}$$ (6)

$$\Phi^{k}=\left[\begin{array}{cc} e^{\eta I_{3}T_{s}} & 0_{3}\\ 0_{3} & e^{-{\left[\omega_{b}\times \right]}^{k}T_{s}} \end{array}\right]$$ (7)

$$Q^{k}\approx \left[\begin{array}{cc} {\sigma_{a}}^{2}{T_{s}}^{2}I_{3} & 0_{3}\\ 0_{3} & {\sigma_{g}}^{2}{T_{s}}^{2}{\left[g_{b}\times \right]}^{k}{\left({\left[g_{b}\times \right]}^{k}\right)}^{T} \end{array}\right]$$ (8)

$$g_{b}=\left[\begin{array}{c} g_{xb}\\ g_{yb}\\ g_{zb} \end{array}\right]$$ (9)

$$\left[g_{b}\times \right]=\left[\begin{array}{ccc} 0 & -g_{zb} & g_{yb}\\ g_{zb} & 0 & -g_{xb}\\ -g_{yb} & g_{xb} & 0 \end{array}\right]$$ (10)

The observation variables are defined as the accelerometer readings, which are the sum of linear and gravitational acceleration, as shown in Eq. 11. Here, **υ** is the measurement error of the accelerometer and is assumed to have zero bias and SD σ_υ_

$$Y=f_{b}=g_{b}-a_{b}+\upsilon$$ (11)

The observation equation for the Kalman filter is then established as shown in Eqs. 12 and 13. The covariance matrix **R***^k^* of **υ** can be derived from Eq. 14 with zero non-diagonal elements, with the simplification that both the process errors and measurement errors are uncorrelated with each other

$$Y^{k}=C^{k}X^{k}+\upsilon^{k}$$ (12)

$$C^{k}=\left[-I_{3}I_{3}\right]$$ (13)

$$R^{k}=\upsilon^{k}{\left(\upsilon^{k}\right)}^{T}$$ (14)

The implementation process of the Kalman filter is shown in fig. S2, where **P**^(*k*+1)/*k*^ is the state vector prediction error, **P***^k^* is the error of the filter output in the *k*th iteration, and **K**^*k*+1^ is the filter gain. Using the gravitational acceleration values outputted by the Kalman filter, the absolute attitude angles of the head (roll angle γ and pitch angle θ) can be calculated using Eqs. 15 and 16.

$$\gamma ={\text{tan}}^{-1}\left(\frac{g_{yb}}{g_{zb}}\right)$$ (15)

$$\theta =-{\text{tan}}^{-1}\left[\frac{g_{xb}}{g_{zb}}\text{cos}\left(\gamma \right)\right]$$ (16)

Calculation of the absolute attitude is independent of the direction in the lateral plane *x*_n_-*y*_n_. To simplify our analysis, we assume that the head direction coincides with geomagnetic north. Thus, the rotation matrix $M_{n}^{b}$ from *x*_n_-*y*_n_-*z*_n_ to *x*_b_-*y*_b_-*z*_b_ can be obtained using Eq. 17.

$$M_{n}^{b}=\left[\begin{array}{ccc} \text{cos}\theta & 0 & -\text{sin}\theta \\ \text{sin}\gamma \text{sin}\theta & \text{cos}\gamma & \text{sin}\gamma \text{cos}\theta \\ \text{cos}\gamma \text{sin}\theta & -\text{sin}\gamma & \text{cos}\gamma \text{cos}\theta \end{array}\right]$$ (17)

The rotation matrix from the head *x*_bh_-*y*_bh_-*z*_bh_ to the lip *x*_bl_-*y*_bl_-*z*_bl_ and chin *x*_bc_-*y*_bc_-*z*_bc_ can then be derived according to Eqs. 18 and 19, respectively, ignoring the directional changes of the lip and chin versus the head

$$M_{h}^{l}={\left(M_{n}^{\text{bh}}\right)}^{-1}M_{n}^{\text{bl}}$$ (18)

$$M_{h}^{c}={\left(M_{n}^{\text{bh}}\right)}^{-1}M_{n}^{\text{bc}}$$ (19)

Last, the relative attitude angles of the lip and chin versus the head are calculated using Eqs. 20 to 23, respectively. Further, the relative angular velocities of the lip (**ω**_L_) and chin (**ω**_C_) are calculated using Eqs. 24 and 25, where **ω**_bh_, **ω**_bl_, and **ω**_bc_ are the angular velocities of the head, lip, and chin in *x*_bh_-*y*_bh_-*z*_bh_, *x*_bl_-*y*_bl_-*z*_bl_ and *x*_bc_-*y*_bc_-*z*_bc_, respectively

$$\theta_{L}=-{\text{tan}}^{-1}\left[\frac{M_{h}^{l}\left(1,3\right)}{M_{h}^{l}\left(1,1\right)}\right]$$ (20)

$$\gamma_{L}=-{\text{tan}}^{-1}\left[\frac{M_{h}^{l}\left(3,2\right)}{M_{h}^{l}\left(2,2\right)}\right]$$ (21)

$$\theta_{C}=-{\text{tan}}^{-1}\left[\frac{M_{h}^{c}\left(1,3\right)}{M_{h}^{c}\left(1,1\right)}\right]$$ (22)

$$\gamma_{C}=-{\text{tan}}^{-1}\left[\frac{M_{h}^{c}\left(3,2\right)}{M_{h}^{c}\left(2,2\right)}\right]$$ (23)

$$\omega_{L}=M_{n}^{\text{bh}}{\left(M_{n}^{\text{bl}}\right)}^{-1}\omega_{\text{bl}}-\omega_{\text{bh}}$$ (24)

$$\omega_{C}=M_{n}^{\text{bh}}{\left(M_{n}^{\text{bc}}\right)}^{-1}\omega_{\text{bc}}-\omega_{\text{bh}}$$ (25)

### Speech detection

We define the speech activity indicator as the geometric mean of the relative angular velocities of the lip, as shown in Eq. 26. A smooth window with a width of 35 sampling periods is adopted, with a moving step of 1 sampling period. The mean value ${\bar{\omega }}_{\text{ind}}$ of the 35 samples during the window is used for speech detection, applying a threshold-based method. The threshold is empirically determined as 2.5% of the maximum value of ${\bar{\omega }}_{\text{ind}}$ during all the speech experiments for each person. In addition, a length threshold of 50 sampling periods is used to eliminate short movements of the lip/chin, which are always generated by nonspeaking movements. Thus, the movement information related to speech is distinguished from that of nonspeech and extracted as speech data

$$\omega_{\text{ind}}=\sqrt{{\omega_{Lx}}^{2}+{\omega_{Ly}}^{2}+{\omega_{Lz}}^{2}}$$ (26)

### Continuous and fluent lip speech movement reconstruction

We have found that the lip speech movement waveforms (attitude angles) keep their main temporal-spatial features during natural speech, whether the word is spoken independently or as part of a continuous sentence. Therefore, we propose a strategy for speech movement reconstruction to artificially synthesize lip speech data from independent words to produce continuous sentences based on our lip language interface. As shown in Figs. 1C and 2 (C to E), there are four steps toward producing continuous sentences from independent words, in the context of the mechanical movement of lip speech:

Step 1: Segment the independent word waveforms (20 samples each word) at the lead and end with appropriate length ratio parameters, as shown in Fig. 2D. The segmentation parameters depend on the natural speaking speed and mannerisms of the person.

Step 2: Concatenate the segmented word waveforms one by one according to their appearance during continuous speech (Figs. 1C and 2C) to generate artificial drafts of continuous speech waveforms (20 samples per sentence).

Step 3: Smooth the drafts using a low-pass filter with cutoff frequency of 8 Hz to generate a natural transition between words (Figs. 1C and 2C) and produce artificial samples of continuous speech (20 samples per sentence).

Step 4: Considering the potential fluctuations of speaking speed, rhythm, and differences in mannerisms between people, data augmentation is conducted, as shown in Fig. 2E. For each independent word sample in step 1, 100 pairs of segmentation parameters are randomly generated around their optimal values by a Monte Carlo method with an SD of 20%. Thus, 100 samples with different segmentations are generated from each original word sample. At the same time, each generated word sample changes its length and magnitude randomly around the original values via a Monte Carlo method with an SD of 20%. By repeating steps 2 and 3 using the generated word samples, 2000 augmented samples are achieved for each sentence.

In this way, we reconstruct the movement waveforms of the lip and chin for naturally spoken sentences by concatenating the related words’ waveforms with proper segmentation at the lead and end. Data augmentation is conducted here to enhance generalizability.

### Bayesian optimization for segmentation parameter recognition

Bayesian optimization provides an efficient tool for globally optimizing objective functions that lack analytic derivatives or are expensive to calculate. By estimating the posterior distribution of the objective function using prior knowledge of the observations of the optimization variables and the corresponding objective function outputs, it predicts the next points to evaluate and allows efficient convergence to the global optimum value (*34*–*36*).

We propose a method on the basis of Bayesian optimization to recognize the segmentation parameters while reconstructing full-sentence waveforms from individual words, as shown in Fig. 2C. The objective function is defined as the similarity between the synthesized and actual sentences and is scaled by custom DTW distance. DTW gives the minimal cumulative distance between two sequences with different lengths, which represents their similarity (*38*–*40*). Here, the synthesized sentence **S**_syn_ and the actual sentence **S**_act_ are two sequences with four channels (rows) and different lengths (columns, *n* and *m*, respectively). The time step $S_{\text{syn}}^{i}$ and $S_{\text{act}}^{j}$ are shown in Eqs. 27 and 28. The iteration of DTW is shown in Eq. 29, where *i* increases from 1 to *n* and *j* increases from 1 to *m*, *d*(*i*, *j*) is the Euclidean distance between $S_{\text{syn}}^{i}$ and $S_{\text{act}}^{j}$ , and *D*(*i*, *j*) is the minimal cumulative distance from step 1 to *i* in **S**_syn_ and from step 1 to *j* in **S**_act_. The normalized DTW is lastly used to evaluate the similarity between **S**_syn_ and **S**_act_ and is adopted as the objective function *f*(**x**) for Bayesian optimization (Eq. 30), where **x** = [*r*_l1_, *r*_e1_, *r*_l2_, *r*_e2_, …, *r*_lN_, *r*_eN_] is the vector of segmentation parameters of all related words contained in **S**_syn_

$$S_{\text{syn}}^{i}={\left[\begin{array}{c} \theta_{L}\\ \gamma_{L}\\ \theta_{C}\\ \gamma_{C} \end{array}\right]}_{\text{syn}}^{i}$$ (27)

$$S_{\text{act}}^{j}={\left[\begin{array}{c} \theta_{L}\\ \gamma_{L}\\ \theta_{C}\\ \gamma_{C} \end{array}\right]}_{\text{act}}^{j}$$ (28)

$$D\left(i,j\right)=d\left(i,j\right)+\text{min}\left[D\left(i-1,j\right),D\left(i,j-1\right),D\left(i-1,j-1\right)\right]$$ (29)

$$f\left(x\right)=\frac{D\left(n,m\right)}{n+m}$$ (30)

This Bayesian optimization process was implemented in MATLAB. Expected improvement (EI) is selected as the acquisition function, which naturally balances exploration and exploitation. The Bayesian optimization is initialized with eight random selected vectors of **x** to give the initial prior distribution knowledge of *f*(**x**). After initial evaluation, Bayesian optimization estimates the posterior distribution while checking a new **x** based on the prior knowledge and selects the new **x** as the next point in the search space by maximizing EI. Here, *f*(**x**) is modeled using a Gaussian process with Gaussian noise $\varepsilon ∼N\left(0,\sigma_{n}^{2}\right)$ . At the *t*th iteration of optimization, a dataset of segmentation parameters and the corresponding DTW distance between the synthesized and actual sentences is attained, **D** = {**X**, **y**}, where **X** = [**x**_1_, **x**_2_, ..., **x**_*t*_]^T^ ∈ R^*t*×2N^, **y** = [*y*_1_, *y*_2_, …, *y_t_*]^T^ ∈ **R**^*t*^, *y_t_* = *f*(**x***_t_*). The prior distribution can be described by mean **m**(**X**) and covariance **K**(**X**, **X**). Without loss of generality, **m**(**X**) = **0**. In addition, *K_ij_* = *k*(**x***_i_*, **x***_j_*) uses ARD Matern5/2 as the kernel function. For a new point to be checked x_*_, the posterior distribution of *f*(x_*_) can be calculated on the basis of the prior distribution and the dataset **D**. The mean and variance of the posterior at x_*_ can be derived from Eqs. 31 and 32, respectively, where k_*_ = [*k*(**x**_1_, x_*_), ..., *k*(**x***_t_*, x_*_)].

$$\mu \left(x_{*}\right)=k_{*}^{T}{\left(K+\sigma_{n}^{2}I\right)}^{-1}y$$ (31)

$$\sigma^{2}\left(x_{*}\right)=k\left(x_{*},x_{*}\right)-k_{*}^{T}{\left(K+\sigma_{n}^{2}I\right)}^{-1}k_{*}$$ (32)

Then, x_*_ is selected as the next point **x**_*t*+1_ by maximizing EI, which is the expected reduction in DTW distance over the smallest value observed previously, EI(x_*_) = max [*f*_min_ − *f*(x_*_),0]. As shown in Eq. 33, x_*_ is determined by the equivalent form of EI, where χ is the search space of **x**, *f*_min_ = min_*i*=1, ..., *t*_E[*f*(**x***_i_*)], Φ(·) and ϕ(·) are the cumulative distribution function and probability density function of the normal distribution, respectively

$$\begin{array}{c} x_{*}={\text{argmax}}_{x_{*}\in \chi }\left[f_{\text{min}}-\mu \left(x_{*}\right)\right]\Phi \left[\frac{f_{\text{min}}-\mu \left(x_{*}\right)}{\sigma \left(x_{*}\right)}\right]+\\ \;\;\sigma \left(x_{*}\right)\phi \left[\frac{f_{\text{min}}-\mu \left(x_{*}\right)}{\sigma \left(x_{*}\right)}\right] \end{array}$$ (33)

Note that a constraint condition is applied during the optimization, where *r*_l*i*_ + *r*_e*i*_ ≤ 1, *i* = 1, …, *N*.

### Word and sentence speech experiments

Eight healthy participants, including four native speakers and four non-native yet proficient speakers, participated in the experiment where just individual words were spoken. As listed in table S1, 93 English words that are commonly used in daily communication including social and emotional contexts and expression of daily needs and in HRI scenarios were selected. During the experiment, the participants sat in front of a computer and spoke the word displayed on the screen. The participants repeated each word 20 times in their natural speaking manner, keeping their mouths idle in-between repetitions in a relaxed, self-selected way. A human operator helped to count and remind the participant when to start/stop the repetition of a word. The speech movements captured by our lip speech interface and the sound of the speakers were recorded at the same time for comparison.

The same eight participants participated in the experiment where whole sentences were spoken. Ninety-three English sentences were selected (table S2), composed of words from the above 93 English words. These sentences are also commonly used in the same five scenarios as in the word speech experiments. The full-sentence speech experiments were conducted following an analogous protocol to that of the individual-word–based speech, except with 10 samples per sentence collected.

### Decoder modeling using TCN-based network

TCN neural networks extract the temporal-spatial features from a time sequence input using a stack of one-dimensional dilated causal convolutional layers. They provide an effective tool for time sequence analysis and classification and are therefore adopted here for lip language decoding. The TensorFlow framework (TensorFlow 2.6) including the built-in Keras framework was used for TCN network modeling. The basic element of the TCN network is the so-called residual block (*41*), which consists of a main path composed of two identical one-dimensional dilated causal convolutional layers and a skip connection that directly adds the input to the output of the main path. The main path is responsible for learning features from the input sequence, and the skip connection facilitates the easier flow of information throughout the network, allowing for better gradient propagation and addressing the vanishing gradient problem (*41*).

For individual word recognition, we developed a TCN-based deep learning neural network (Fig. 3D) to model the decoder. To ensure that the receptive field of the network covered the input sequence length, six such TCN residual blocks were stacked with a kernel size of 2 and dilations of 1, 2, 4, 8, 16, and 32, respectively. Then, a layer of one-dimensional global average pooling was added to further extract features from the TCN’s sequential output. This was then connected to a fully connected layer with the softmax activation function used for classification. The Adam optimizer was used for network training, and the same filter number was shared by all TCN residual blocks. The hyperparameter optimization is shown in fig. S7. Following the optimization result, the filter number was selected as 400. One-dimensional global average pooling and one-dimensional global max pooling and flattening (no pooling) were compared as shown in fig. S7. One-dimensional global average pooling outperformed the others and was therefore selected. *Z*-score normalization and zero padding of the data (to ensure that samples were all of the same length) were conducted for sequence data of individual word speech before being inputted into the network. Tenfold cross-validation tests with randomly mixed datasets were performed, where the 20 datasets, comprising 93 words each, were randomly divided into 10 folds. One to eight folds were increasingly selected for network training, whereas the rest were used to test the network (Fig. 3, C and D). The average recognition accuracy of each cross-validation setup was used in the final evaluation.

Full-sentence speech recognition here is another task independent from word recognition, aiming at evaluating the effectiveness of our speech movement reconstruction strategy to generate continuous and fluent lip speech data and the method of data-efficient continuous and fluent lip language recognition. Therefore, another independent TCN-based neural network (Fig. 4E) was used here for decoder modeling. It had a similar structure to the previously described network used for individual word speech recognition, with the only difference coming in the number of TCN residual blocks used. The sentence speech recognition neural network contained seven TCN residual blocks, with a kernel size of 2 and dilations of 1, 2, 4, 8, 16, 32, and 64, respectively, to cover the comparatively longer input sequence length of the sentences. As before, all the TCN residual blocks shared the same filter number. As shown in fig. S8, the filter number was selected as 400 according to the results of optimization, and a one-dimensional global average pooling was selected because it outperformed one-dimensional global max pooling and flattening (no pooling). Full-sentence speech datasets were created with *z*-score normalization and zero padding. All the artificially generated and augmented samples produced for each sentence were used for network training, and all the actual sentence samples were used for network testing.

### Participants

Participants were given the necessary background information about the experiment and what tasks they should expect to complete. All were healthy and had no medical conditions that would interfere with their ability to speak. The experiments performed for this study involving human participants were approved by the Institution Review Board of the University of Cambridge (no. PRE.2023.063). Informed consent was obtained from the human participants to use their image and conduct the experiments described here.

### Statistics

Statistical analyses were conducted using MATLAB 2019b (MathWorks, USA). All the results related to multiple participants were evaluated using the mean value and the SD.

## References

[1] Q. S. Yang, W. Q. Jin, Q. H. Zhang, Y. H. Wei, Z. F. Guo, X. S. Li, Y. Yang, Q. Q. Luo, H. Tian, T. L. Ren, Mixed-modality speech recognition and interaction using a wearable artificial throat. Nat. Mach. Intell. 5, 169–180 (2023).

[2] Y. Lu, H. Tian, J. Cheng, F. Zhu, B. Liu, S. Wei, L. Ji, Z. L. Wang, Decoding lip language using triboelectric sensors with deep learning. Nat. Commun. 13, 1401 (2022). 35301313 10.1038/s41467-022-29083-0 PMC8931018

[3] T. Kim, Y. Shin, K. Kang, K. Kim, G. Kim, Y. Byeon, H. Kim, Y. Gao, J. R. Lee, G. Son, T. Kim, Y. Jun, J. Kim, J. Lee, S. Um, Y. Kwon, B. G. Son, M. Cho, M. Sang, J. Shin, K. Kim, J. Suh, H. Choi, S. Hong, H. Cheng, H.-G. Kang, D. Hwang, K. J. Yu, Ultrathin crystalline-silicon-based strain gauges with deep learning algorithms for silent speech interfaces. Nat. Commun. 13, 5815 (2022). 36192403 10.1038/s41467-022-33457-9 PMC9530138

[4] T. Schultz, M. Wand, T. Hueber, D. J. Krusienski, C. Herff, J. S. Brumberg, Biosignal-based spoken communication: A survey. IEEE/ACM Trans. Audio Speech Lang. 25, 2257–2271 (2017).

[5] K. Sun, C. Yu, W. Shi, L. Liu, Y. Shi, in *31st Annual ACM Symposium on User Interface Software and Technology (UIST)* (Association for Computing Machinery, 2018), pp. 581–593.

[6] Y. M. Assael, B. Shillingford, S. Whiteson, N. De Freitas, Lipnet: End-to-end sentence-level lipreading. arXiv:1611.01599 (2016).

[7] P. Singari, C. Naganoshith, S. Mehta, K. S. Rao, Y. BM, Deciphering lip movement a comparative study. Int. J. Eng. Res. Technol. 12, 10.17577/IJERTV12IS060128 (2023).

[8] M. J. Fagan, S. R. Ell, J. M. Gilbert, E. Sarrazin, P. M. Chapman, Development of a (silent) speech recognition system for patients following laryngectomy. Med. Eng. Phys. 30, 419–425 (2008). 17600751 10.1016/j.medengphy.2007.05.003

[9] J. M. Gilbert, S. I. Rybchenko, R. Hofe, S. R. Ell, M. J. Fagan, R. K. Moore, P. Green, Isolated word recognition of silent speech using magnetic implants and sensors. Med. Eng. Phys. 32, 1189–1197 (2010). 20863739 10.1016/j.medengphy.2010.08.011

[10] J. A. Gonzalez, L. A. Cheah, A. M. Gomez, P. D. Green, J. M. Gilbert, S. R. Ell, R. K. Moore, E. Holdsworth, Direct speech reconstruction from articulatory sensor data by machine learning. IEEE/ACM Trans. Audio Speech Lang. 25, 2362–2374 (2017).

[11] T. Hueber, E. L. Benaroya, G. Chollet, B. Denby, G. Dreyfus, M. Stone, Development of a silent speech interface driven by ultrasound and optical images of the tongue and lips. Speech Commun. 52, 288–300 (2010).

[12] N. Kimura, M. Kono, J. Rekimoto, in *CHI Conference on Human Factors in Computing Systems (CHI)* (Association for Computing Machinery, 2019), pp 1–11.

[13] P. Birkholz, S. Stone, K. Wolf, D. Plettemeier, Non-invasive silent phoneme recognition using microwave signals. IEEE/ACM Trans. Audio Speech Lang. 26, 2404–2411 (2018).

[14] D. Ferreira, S. Silva, F. Curado, A. Teixeira, Exploring silent speech interfaces based on frequency-modulated continuous-wave radar. Sensors 22, 649 (2022). 35062610 10.3390/s22020649 PMC8781659

[15] H. Liu, W. Dong, Y. Li, F. Li, J. Geng, M. Zhu, T. Chen, H. Zhang, L. Sun, C. Lee, An epidermal sEMG tattoo-like patch as a new human-machine interface for patients with loss of voice. Microsyst. Nanoeng. 6, 16 (2020). 34567631 10.1038/s41378-019-0127-5 PMC8433406

[16] J. Kwon, H. Nam, Y. Chae, S. Lee, I. Y. Kim, C.-H. Im, Novel three-axis accelerometer-based silent speech interface using deep neural network. Eng. Appl. Artif. Intel. 120, 105909 (2023).

[17] T. Schultz, M. Wand, Modeling coarticulation in EMG-based continuous speech recognition. Speech Commun. 52, 341–353 (2010).

[18] M. Janke, L. Diener, EMG-to-speech: Direct generation of speech from facial electromyographic signals. IEEE/ACM Trans. Audio Speech Lang. 25, 2375–2385 (2017).

[19] M. Zhu, H. Zhang, X. Wang, X. Wang, Z. Yang, C. Wang, O. W. Samuel, S. Chen, G. Li, Towards optimizing electrode configurations for silent speech recognition based on high-density surface electromyography. J. Neural Eng. 18, 016005 (2021). 10.1088/1741-2552/abca14 33181497

[20] G. S. Meltzner, J. T. Heaton, Y. Deng, G. De Luca, S. H. Roy, J. C. Kline, Development of sEMG sensors and algorithms for silent speech recognition. J. Neural Eng. 15, 046031 (2018). 29855428 10.1088/1741-2552/aac965 PMC6168082

[21] J. Rekimoto, Y. Nishimura, in *Augmented Humans Conference (AHs)* (Association for Computing Machinery, 2021), pp. 91–100.

[22] C. J. De Luca, The use of surface electromyography in biomechanics. J. Appl. Biomech. 13, 135–163 (1997).

[23] M. Cifrek, V. Medved, S. Tonkovic, S. Ostojic, Surface EMG based muscle fatigue evaluation in biomechanics. Clin. Biomech. 24, 327–340 (2009). 10.1016/j.clinbiomech.2009.01.010 19285766

[24] S. Q. Liu, J. C. Zhang, G. Z. Li, R. Zhu, A wearable flow-MIMU device for monitoring human dynamic motion. IEEE Trans. Neural Syst. Rehabil. Eng. 28, 637–645 (2020). 32031941 10.1109/TNSRE.2020.2971762

[25] Y. Tian, H. Wei, J. Tan, An adaptive-gain complementary filter for real-time human motion tracking with MARG sensors in free-living environments. IEEE Trans. Neural Syst. Rehabil. Eng. 21, 254–264 (2013). 22801527 10.1109/TNSRE.2012.2205706

[26] D. Bandanau, J. Chorowski, D. Serdyuk, P. Brakel, Y. Bengio, in *41st IEEE International Conference on Acoustics, Speech and Signal Processing (ICASSP)* (IEEE, 2016), pp. 4945–4949.

[27] D. Norris, J. M. McQueen, Shortlist B: A Bayesian model of continuous speech recognition. Psychol. Rev. 115, 357–395 (2008). 18426294 10.1037/0033-295X.115.2.357

[28] I. Sheikh, E. Vincent, I. Illina, Training RNN language models on uncertain ASR hypotheses in limited data scenarios. Comput. Speech Lang. 83, 101555 (2024).

[29] G. E. Dahl, D. Yu, L. Deng, A. Acero, Context-dependent pre-trained deep neural networks for large-vocabulary speech recognition. IEEE Trans. Audio Speech Lang. Process. 20, 30–42 (2012).

[30] R. Zhu, Z. Zhou, A real-time articulated human motion tracking using tri-axis inertial/magnetic sensors package. IEEE Trans. Neural Syst. Rehab. Eng. 12, 295–302 (2004). 10.1109/TNSRE.2004.827825 15218943

[31] S. Liu, J. Zhang, Y. Zhang, R. Zhu, A wearable motion capture device able to detect dynamic motion of human limbs. Nat. Commun. 11, 5615 (2020). 33154381 10.1038/s41467-020-19424-2 PMC7645594

[32] S. Q. Liu, J. C. Zhang, R. Zhu, A wearable human motion tracking device using micro flow sensor incorporating a micro accelerometer. IEEE Trans. Biomed. Eng. 67, 940–948 (2020). 31247541 10.1109/TBME.2019.2924689

[33] E. Palermo, S. Rossi, F. Marini, F. Patane, P. Cappa, Experimental evaluation of accuracy and repeatability of a novel body-to-sensor calibration procedure for inertial sensor-based gait analysis. Measurement 52, 145–155 (2014).

[34] Y. Ding, M. Kim, S. Kuindersma, C. J. Walsh, Human-in-the-loop optimization of hip assistance with a soft exosuit during walking. Sci. Robot. 3, eaar5438 (2018). 33141683 10.1126/scirobotics.aar5438

[35] H. J. Kushner, A new method of locating the maximum point of an arbitrary multipeak curve in the presence of noise. J. Basic Eng. 86, 97–106 (1964).

[36] B. Shahriari, K. Swersky, Z. Wang, R. P. Adams, N. de Freitas, Taking the human out of the loop: A review of bayesian optimization. Proc. IEEE 104, 148–175 (2016).

[37] C. A. Ratanamahatana, E. Keogh, in *Third Workshop on Mining Temporal and Sequential Data*, vol. 32 (Citeseer, 2004), pp. 50–60.

[38] S. E. Anderson, A. S. Dave, D. Margoliash, Template-based automatic recognition of birdsong syllables from continuous recordings. J. Acoust. Soc. Am. 100, 1209–1219 (1996). 8759970 10.1121/1.415968

[39] S. Wu, M. Li, X. Dong, L. Cheng, A. Aljuaid, in *2nd International Conference on Cognitive Based Information Processing and Applications (CIPA 2022)* (Springer, 2022), pp. 155–162.

[40] C. Yu, X. Wang, A russian continuous speech recognition system based on the dtw algorithm under artificial intelligence. J. Robot. 2022, 5777472 (2022).

[41] S. Bai, J. Z. Kolter, V. Koltun, An empirical evaluation of generic convolutional and recurrent networks for sequence modeling. arXiv:1803.01271 (2018).

