# Supplementary material for: A data-efficient and easy-to-use lip language interface based on wearable motion capture and speech movement reconstruction
Source: Sci Adv. 2024 Jun 26;10(26):eado9576. doi: 10.1126/sciadv.ado9576 (PMC11204283; doi:10.1126/sciadv.ado9576)
Supplement: Supplementary file 1 — Figs. S1 to S22 Tables S1 and S2 Legend for table S3 Legends for movies S1 and S2 [file sciadv.ado9576_sm.pdf]

Science Advances
AAAS

Supplementary Materials for

A data-efficient and easy-to-use lip language interface based on wearable motion capture and speech movement reconstruction

Shiqiang Liu *et al.*

Corresponding author: Rong Zhu, zr_gloria@mail.tsinghua.edu.cn; George G. Malliaras, gm603@cam.ac.uk; Manohar Bance, mlb59@cam.ac.uk

Sci. Adv. **10**, eado9576 (2024)
DOI: 10.1126/sciadv.ado9576

The PDF file includes:

Figs. S1 to S22
Tables S1 and S2
Legend for table S3
Legends for movies S1 and S2

Other Supplementary Material for this manuscript includes the following:

Table S3
Movies S1 and S2

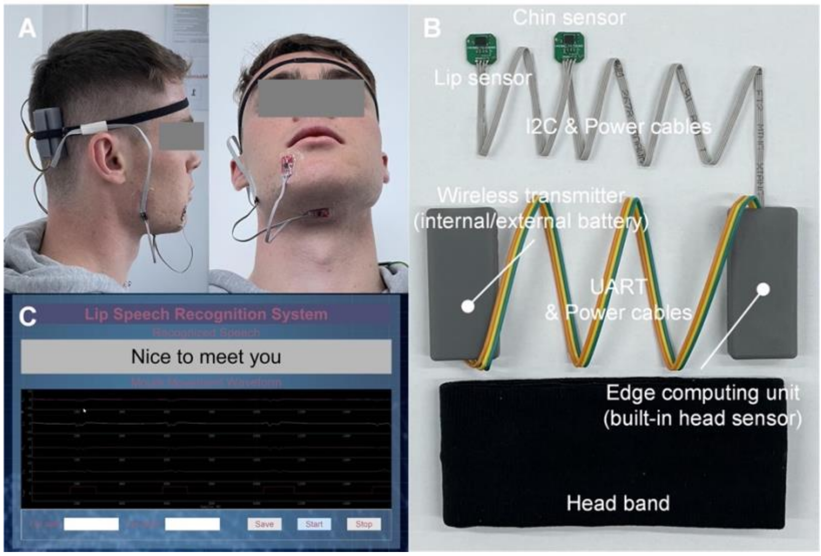

Fig. S1. The developed wearable lip language recognition system.

(**A**) The system worn on a user. (**B**) Components and wire connections of the system. (**C**) The software based on Python running on the host computer.

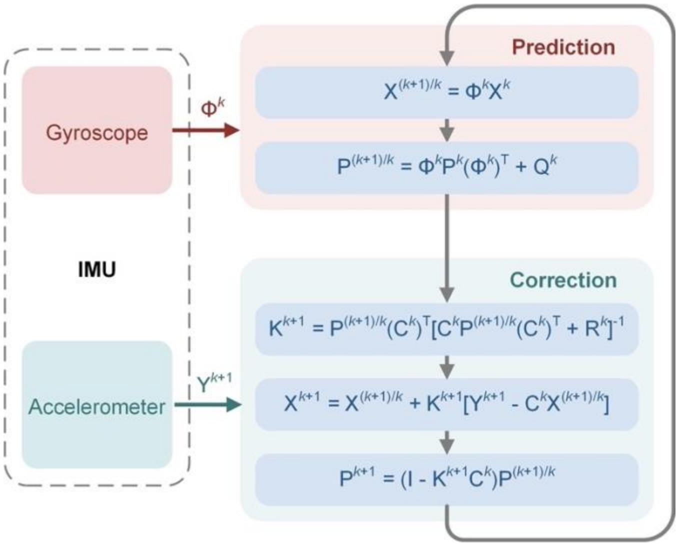

Fig. S2. The Kalman filtering process for data fusion.

$\mathbf{P}^{(k+1)/k}$ is the state vector prediction error, $\mathbf{P}^k$ is the error of the filter output in the *k*th iteration, $\mathbf{K}^{k+1}$ is the filter gain.

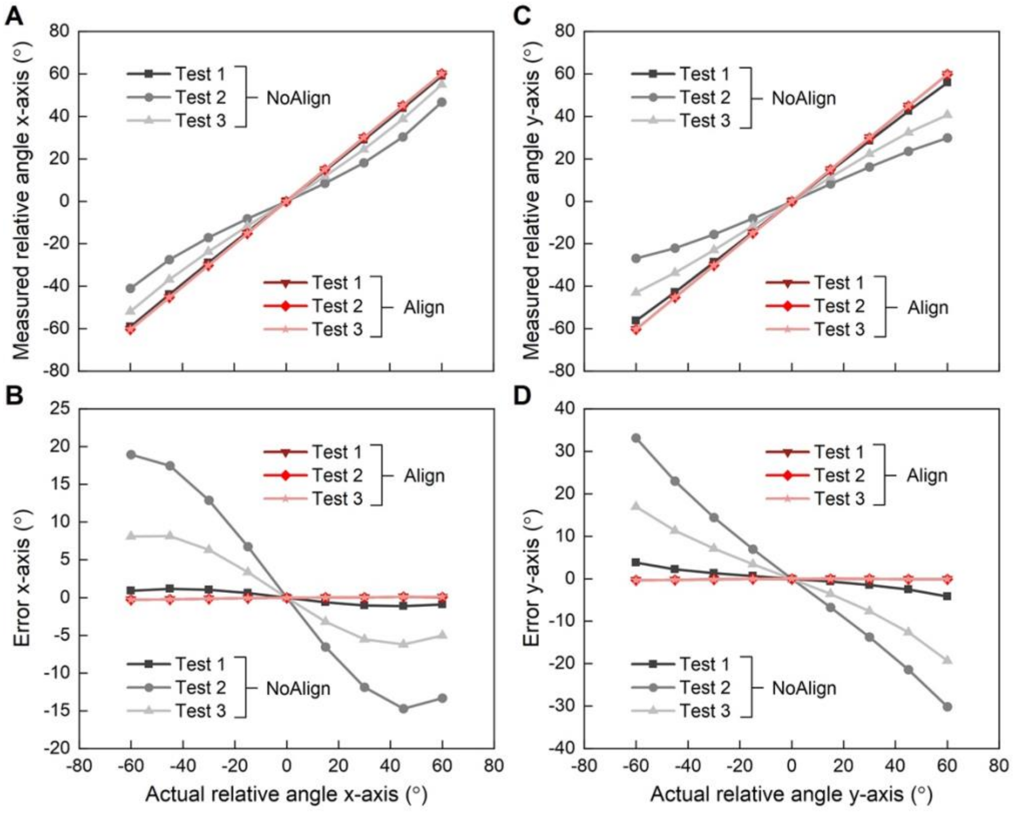

Fig. S3. Experimental results of misalignment elimination of our lip language interface (the chin sensor versus the head sensor).

(**A**) The captured relative movement (rotation angle) of the chin versus the head along x-axis of the head body coordinate system, and (**B**) the corresponding errors before (NoAlign) and after misalignment elimination (Align). (**C**) The captured relative movement (rotation angle) of the chin versus the head along y-axis of the head body coordinate system, and (**D**) the corresponding errors before (NoAlign) and after misalignment elimination (Align). Test 1, 2 and 3 are three independent tests with different misalignments.

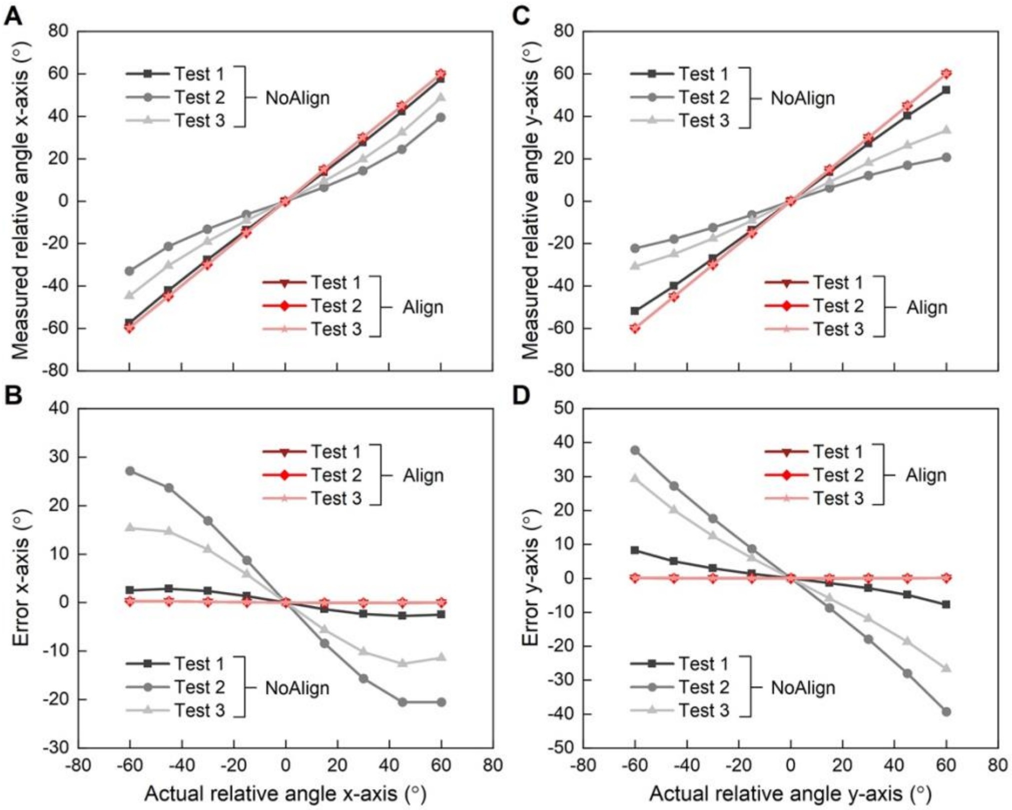

Fig. S4. Experimental results of misalignment elimination of our lip language interface (the lip sensor versus the head sensor).

(**A**) The captured relative movement (rotation angle) of the lip versus the head along x-axis of the head body coordinate system, and (**B**) the corresponding errors before (NoAlign) and after misalignment elimination (Align). (**C**) The captured relative movement (rotation angle) of the lip versus the head along y-axis of the head body coordinate system, and (**D**) the corresponding errors before (NoAlign) and after misalignment elimination (Align). Test 1, 2 and 3 are three independent tests with different misalignments.

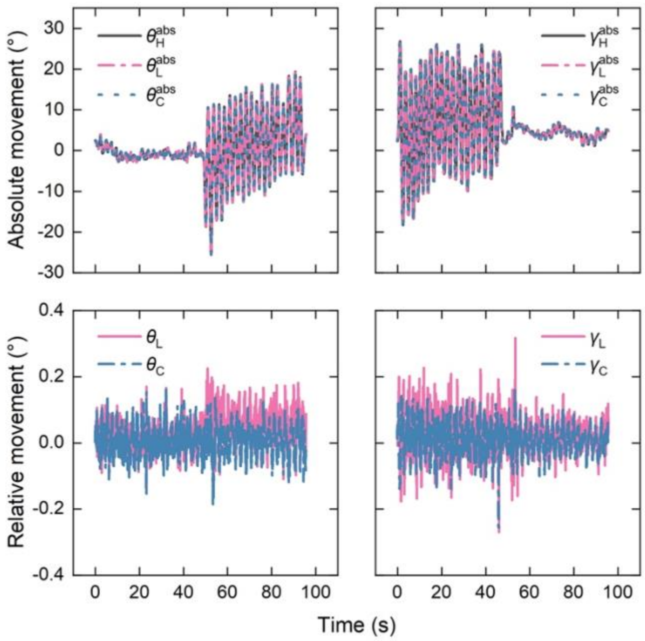

Fig. S5. Experimental results of interference elimination of our lip language interface. $\theta_{\mathrm{H}}^{\mathrm{abs}}$, $\gamma_{\mathrm{H}}^{\mathrm{abs}}$, $\theta_{\mathrm{L}}^{\mathrm{abs}}$, $\gamma_{\mathrm{L}}^{\mathrm{abs}}$, $\theta_{\mathrm{C}}^{\mathrm{abs}}$ and $\gamma_{\mathrm{C}}^{\mathrm{abs}}$ are the absolute attitude angles of the head, lip and chin respectively. θ_{L}, γ_{L}, θ_{C} and γ_{C} refer to the relative attitude angles of the lip and chin versus the head. Because there are no relative movements of the lip sensor and chin sensor versus the head sensor, θ_{L}, γ_{L}, θ_{C} and γ_{C} represent the system errors caused by head movement interference.

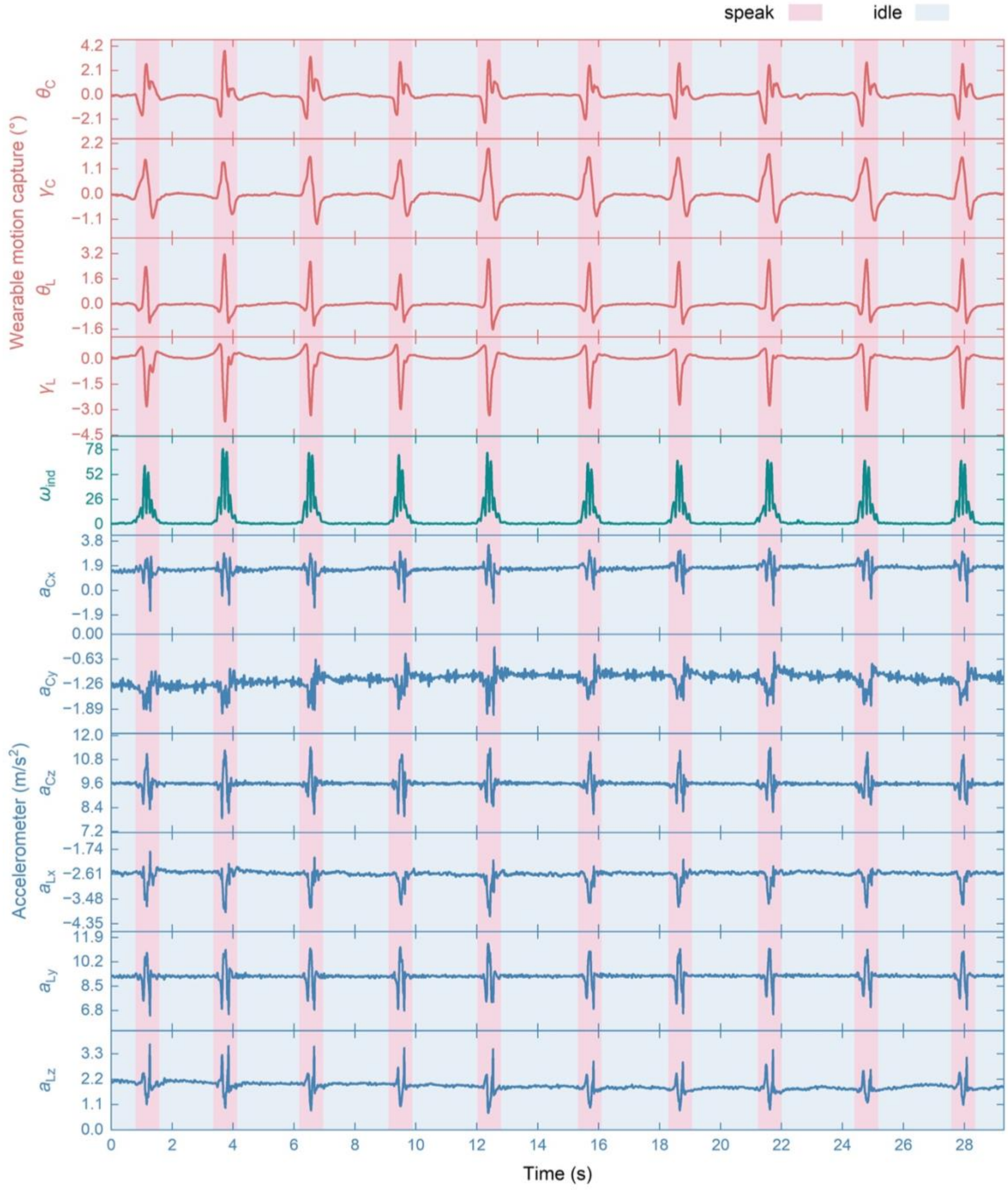

Fig. S6. The captured movement information related to lip language during individual word speech.

θ_{C}, γ_{C}, θ_{L}, and γ_{L} are the relative attitude angles of the lip and chin. ω_{ind} is the angular velocity indicator for speech detection. a_{Cx}, a_{Cy}, a_{Cz}, a_{Lx}, a_{Ly} and a_{Lz} are the raw outputs of the accelerometers on the chin and lip under the sensors' coordinate systems.

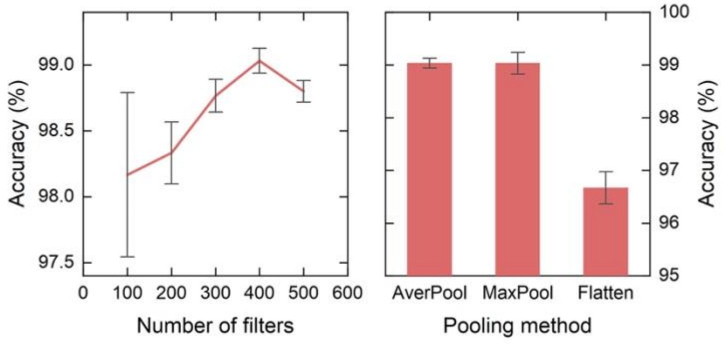

Fig. S7. Hyperparameter optimization for the deep neural network for individual word speech recognition.

The recognition accuracy versus filter number of TCN residual blocks is shown in the left graph. 1-dimensional global average pooling, 1-dimensional global max pooling and flattening (no pooling), which further extract features from TCN's sequence output and then feed the feature vector into the fully connected layer, are compared as shown in the right graph.

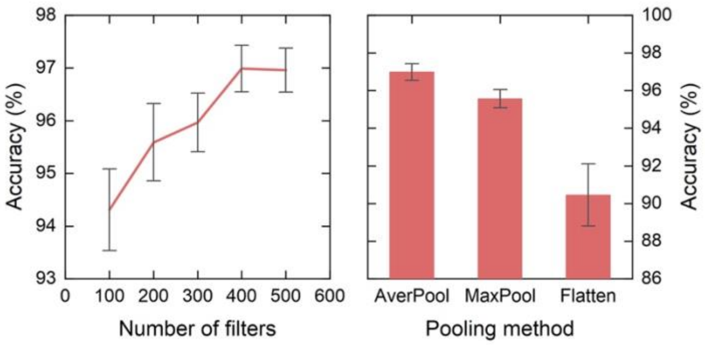

Fig. S8. Hyperparameters optimization for the deep neural network for sentence speech recognition.

The recognition accuracy versus filter number of TCN residual blocks is shown in the left graph. 1-dimensional global average pooling, 1-dimensional global max pooling and flattening (no pooling), which further extract features from the TCN's sequential output and feed the feature vector into the fully connected layer, are compared in the right graph.

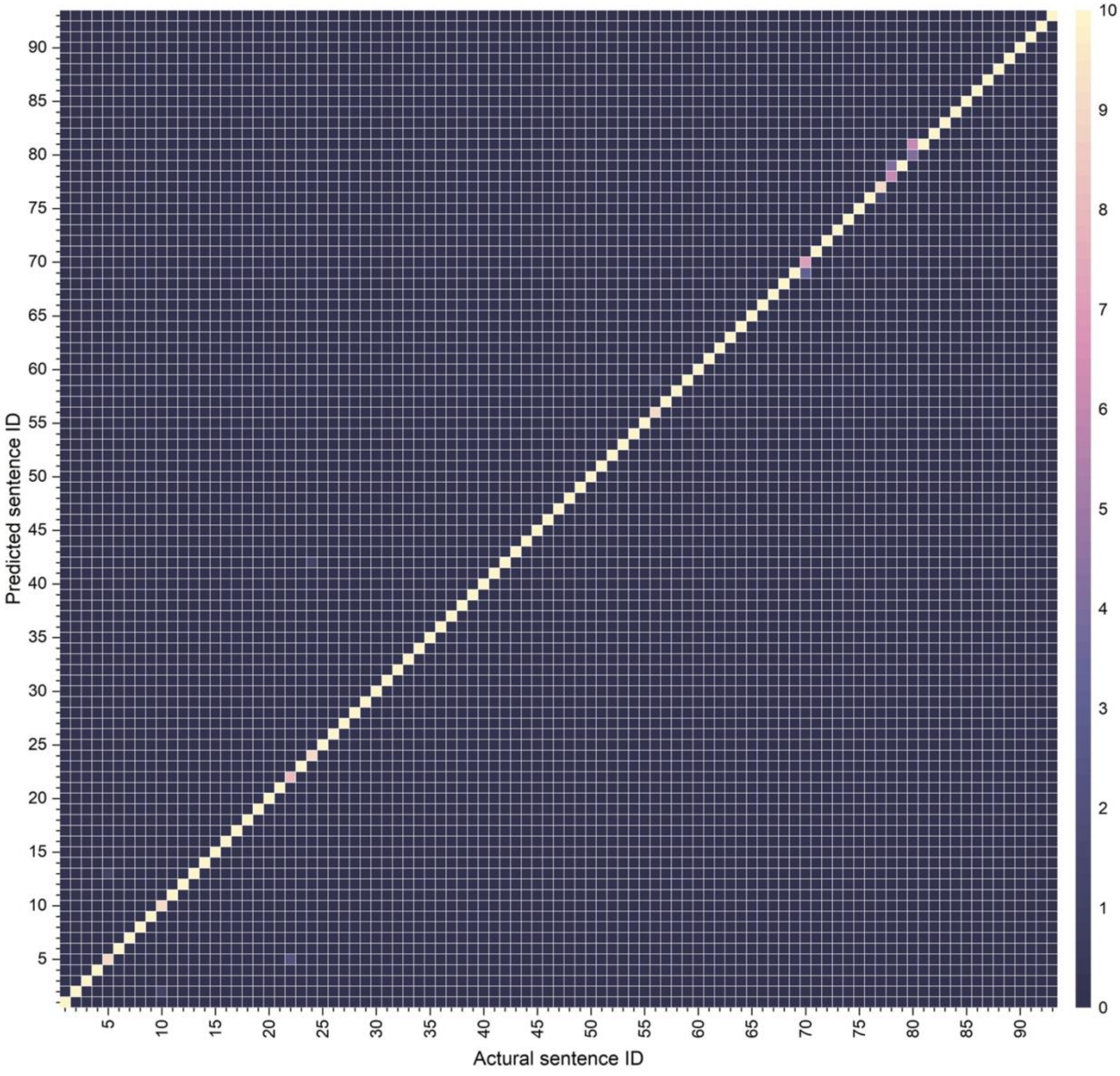

Fig. S9. Recognition accuracy for the 93 sentences, using individual parameters (Sub. 2)

Artificial sentence datasets, produced using the subject's own segmentation parameters, are used for deep neural network training, and all the actual sentence samples are used for recognition testing.

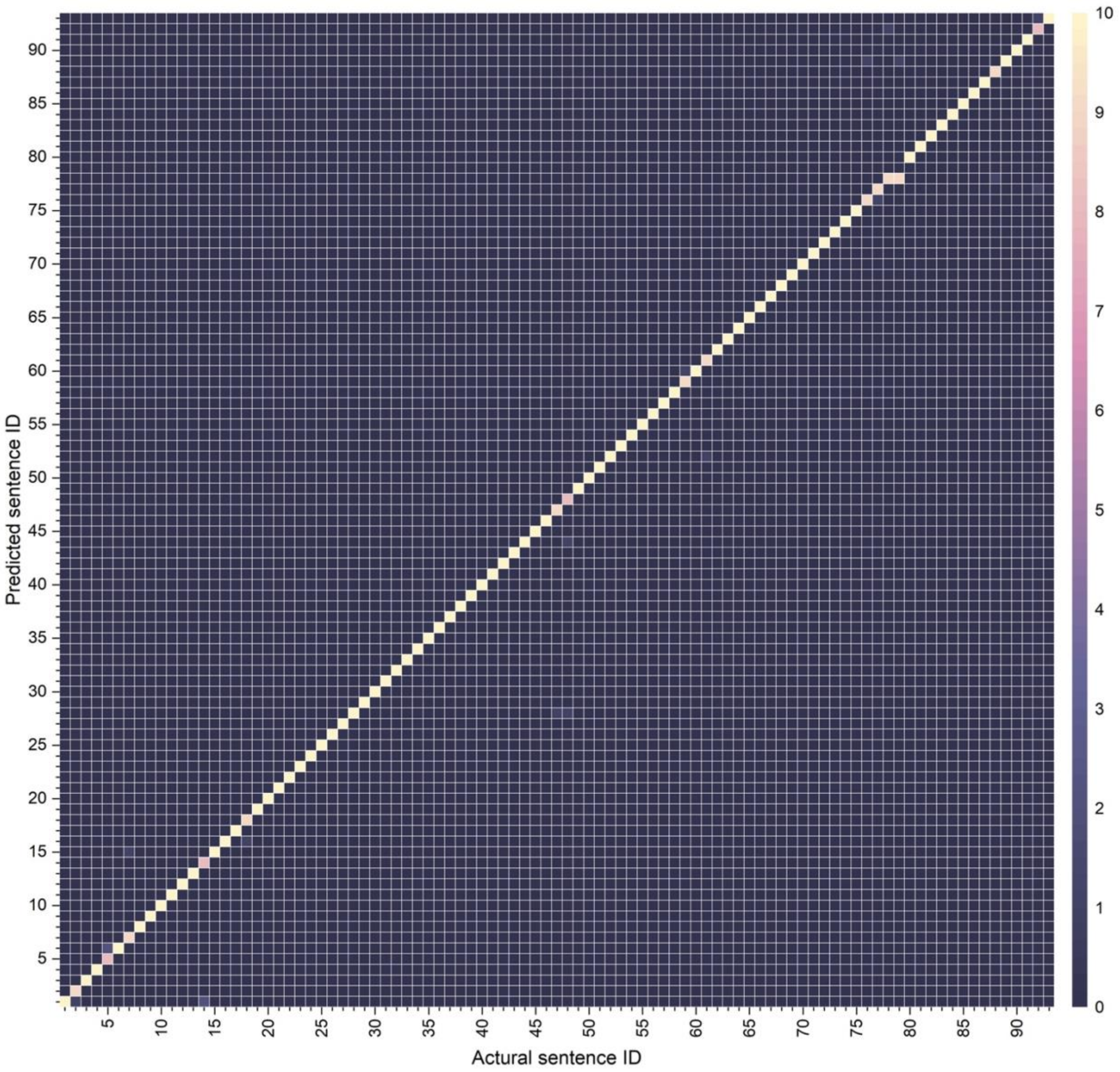

Fig. S10. Recognition accuracy for the 93 sentences, using individual parameters (Sub. 3)

Artificial sentence datasets, produced using the subject's own segmentation parameters, are used for deep neural network training, and all the actual sentence samples are used for recognition testing.

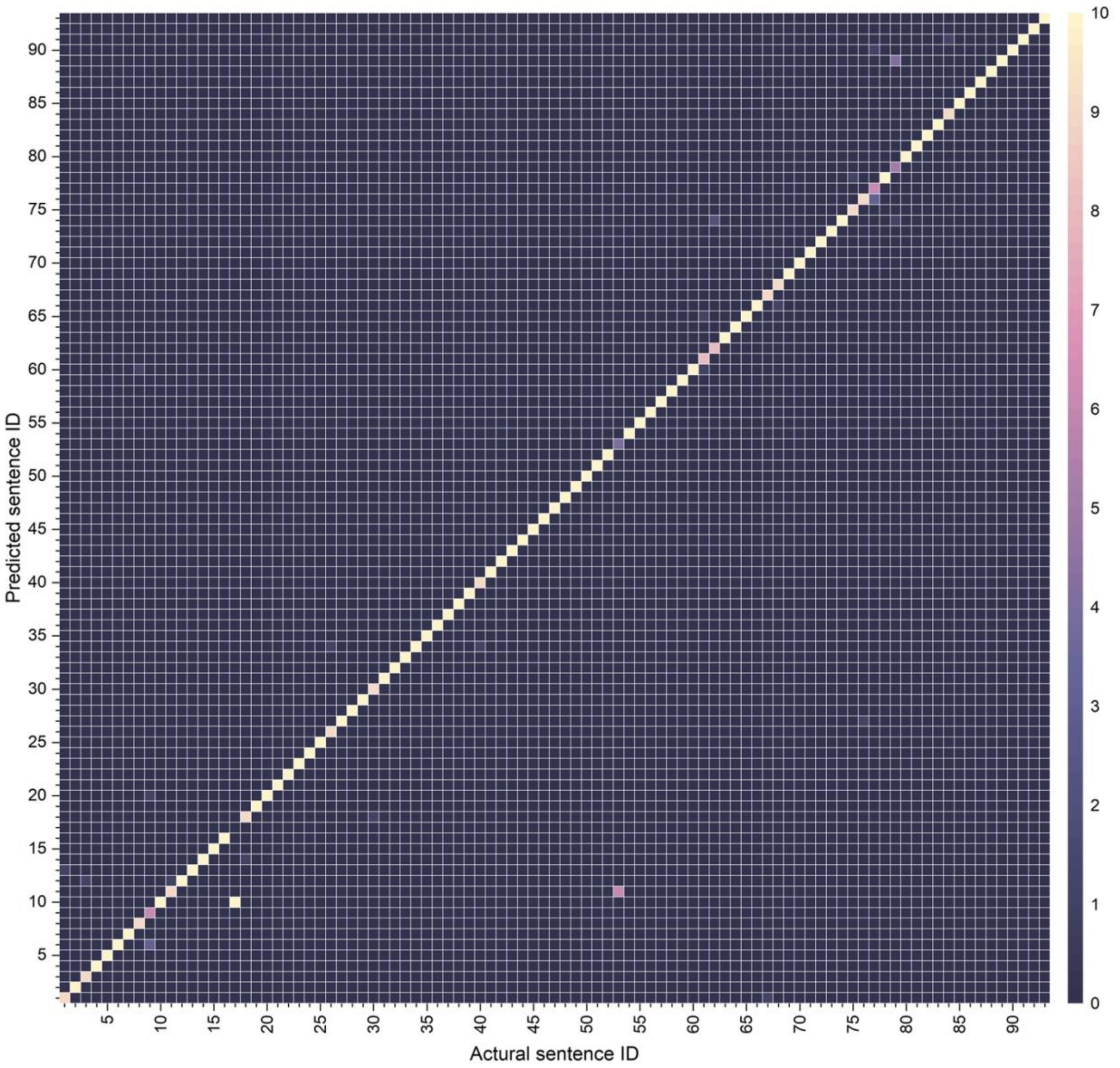

Fig. S11. Recognition accuracy for the 93 sentences, using individual parameters (Sub. 4)

Artificial sentence datasets, produced using the subject's own segmentation parameters, are used for deep neural network training, and all the actual sentence samples are used for recognition testing.

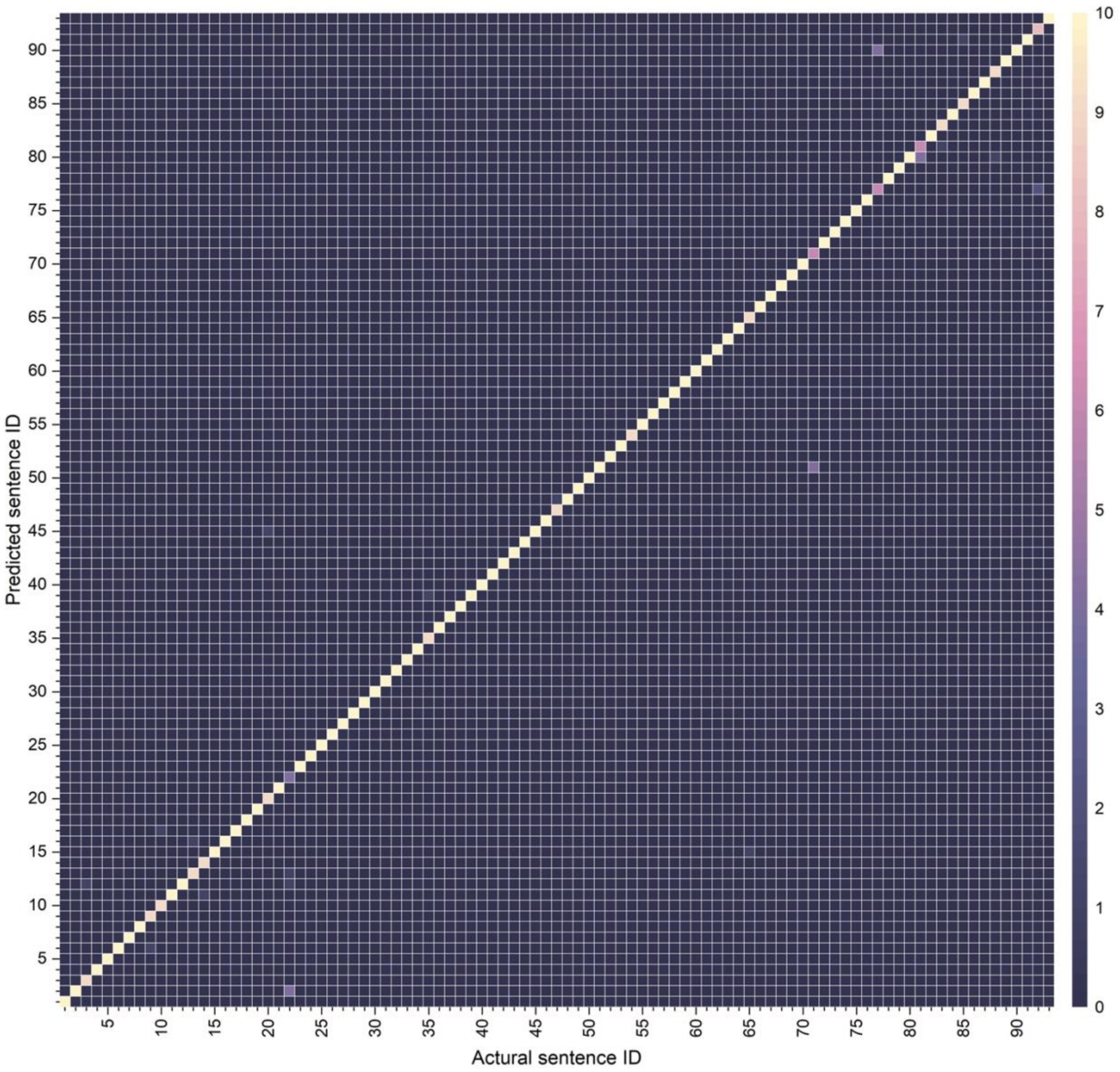

Fig. S12. Recognition accuracy for the 93 sentences, using individual parameters (Sub. 5)

Artificial sentence datasets, produced using the subject's own segmentation parameters, are used for deep neural network training, and all the actual sentence samples are used for recognition testing.

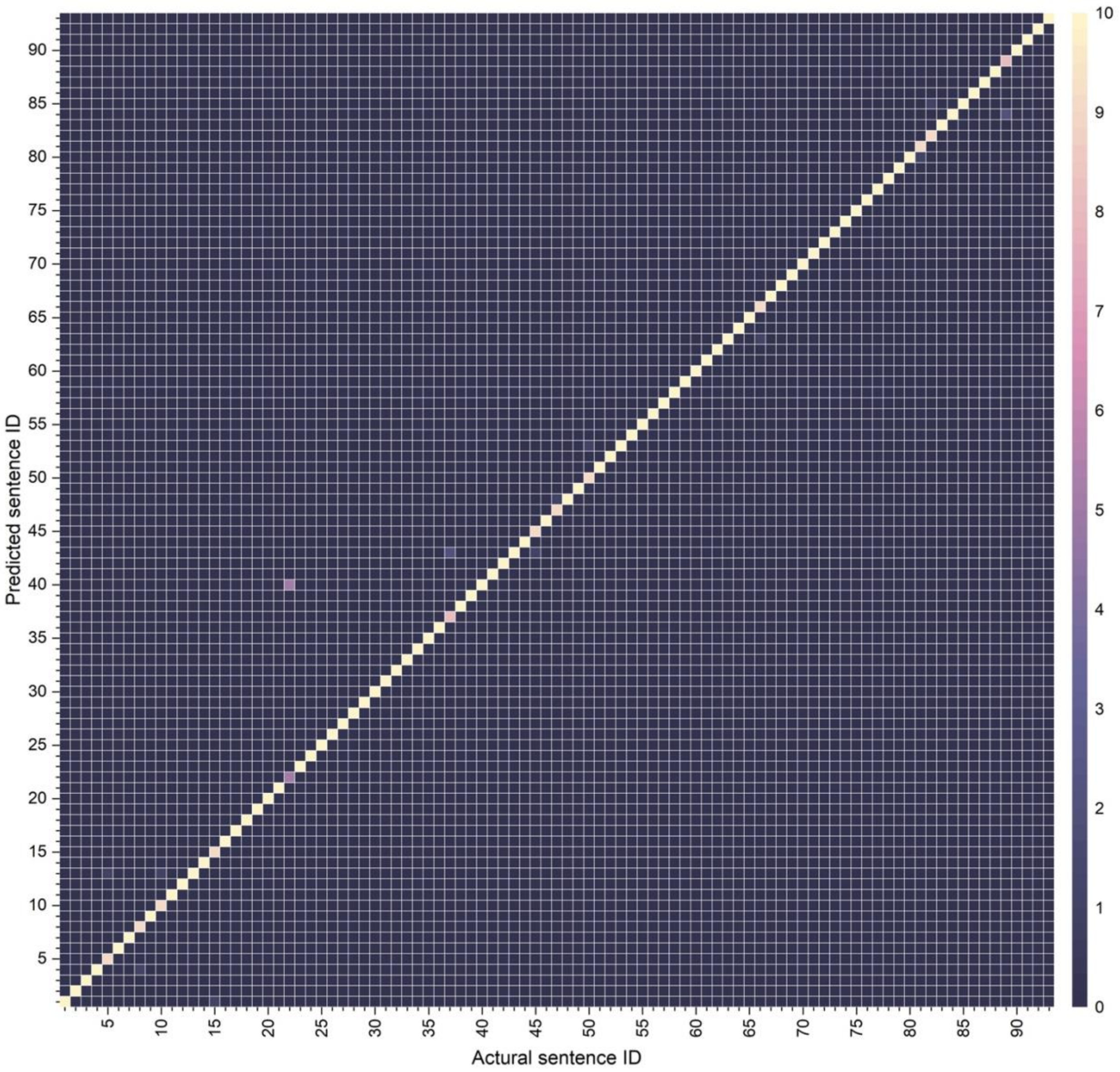

Fig. S13. Recognition accuracy for the 93 sentences, using individual parameters (Sub. 6)

Artificial sentence datasets, produced using the subject's own segmentation parameters, are used for deep neural network training, and all the actual sentence samples are used for recognition testing.

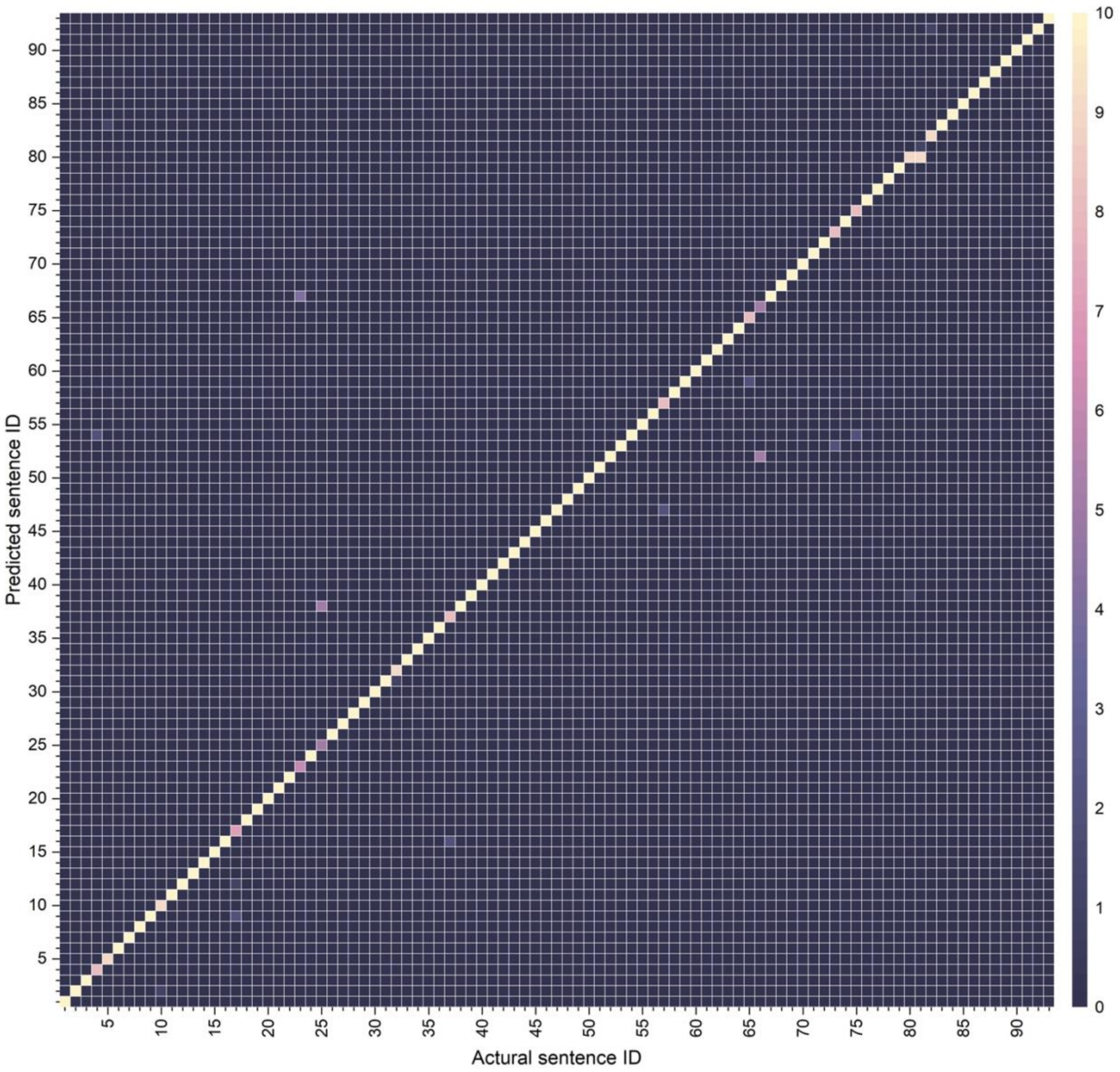

Fig. S14. Recognition accuracy for the 93 sentences, using individual parameters (Sub. 7)

Artificial sentence datasets, produced using the subject's own segmentation parameters, are used for deep neural network training, and all the actual sentence samples are used for recognition testing.

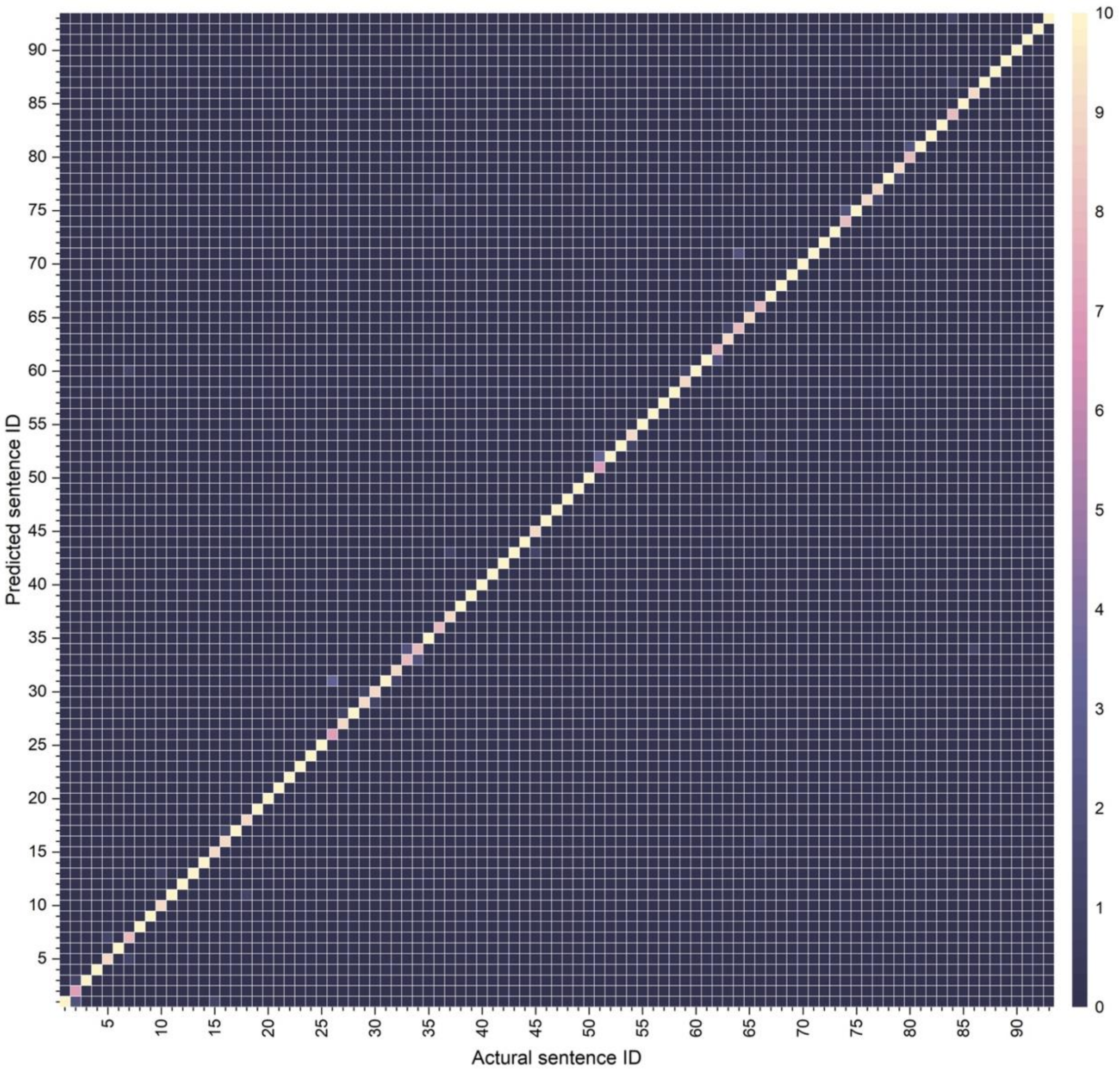

Fig. S15. Recognition accuracy for the 93 sentences, using individual parameters (Sub. 8)

Artificial sentence datasets, produced using the subject's own segmentation parameters, are used for deep neural network training, and all the actual sentence samples are used for recognition testing.

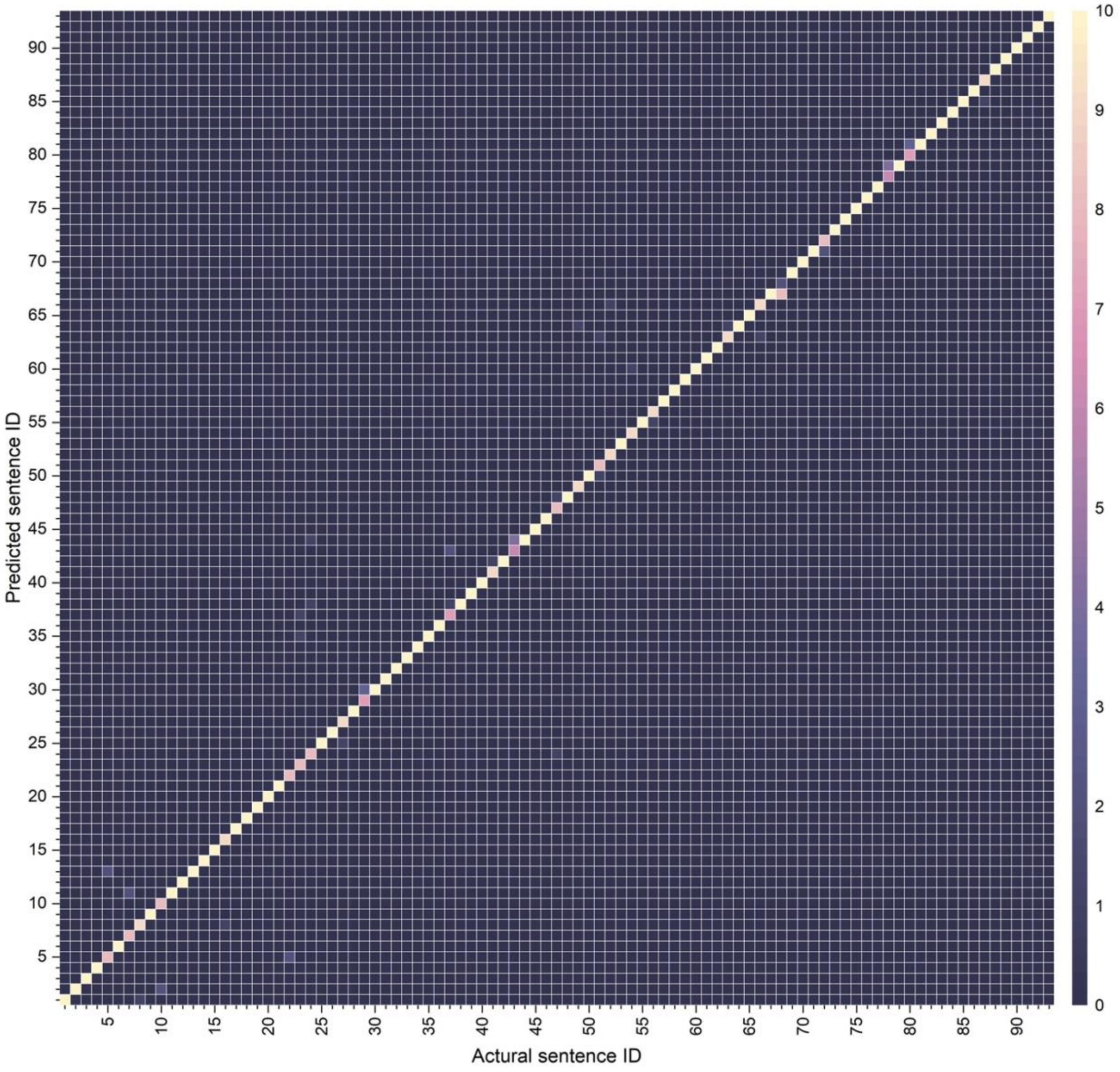

Fig. S16. Recognition accuracy for the 93 sentences, using general parameters (Sub. 2)

Artificial sentence datasets, produced using general segmentation parameters from Sub. 1, are used for deep neural network training, and all the actual sentence samples are used for recognition testing.

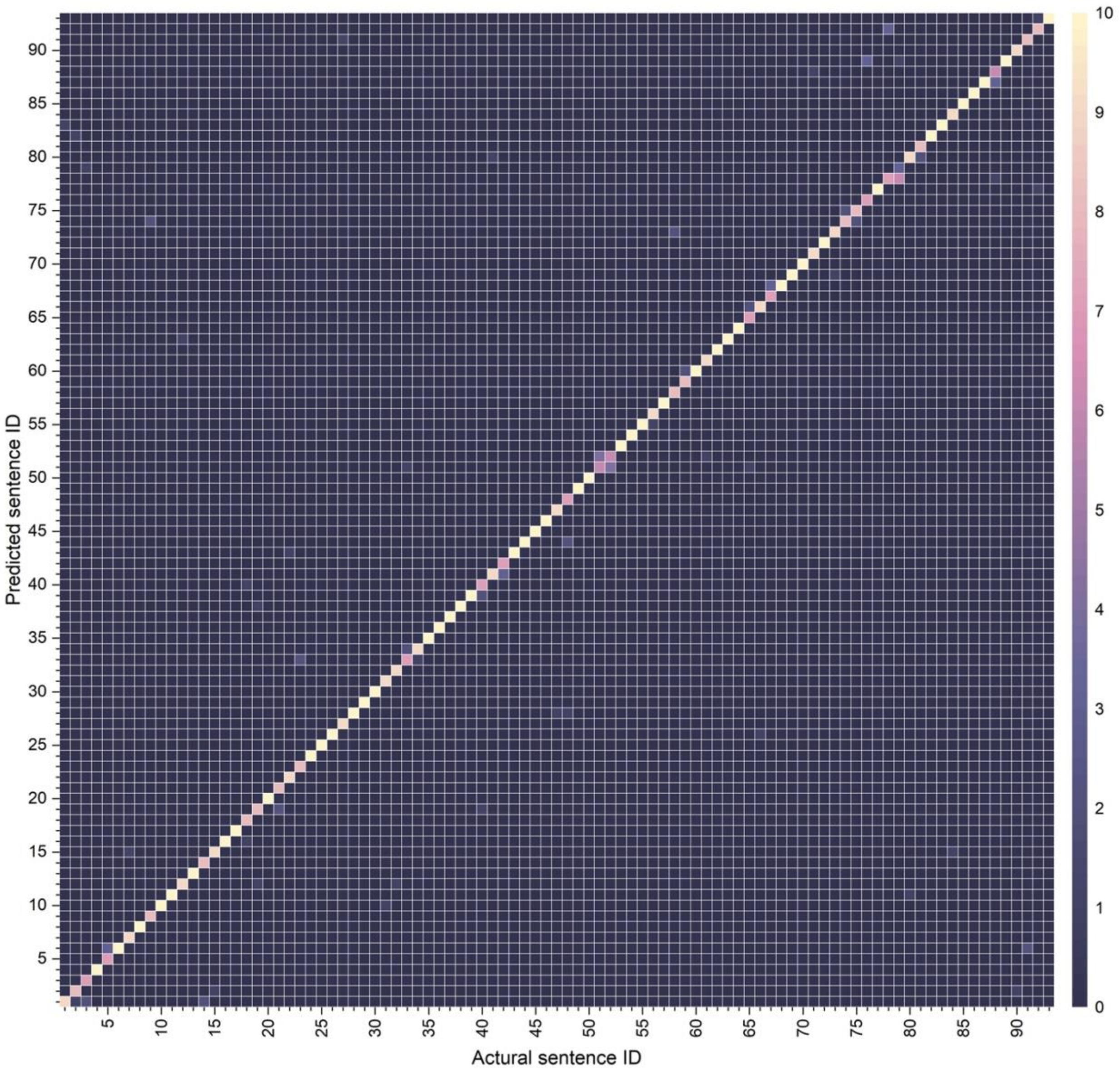

Fig. S17. Recognition accuracy for the 93 sentences, using general parameters (Sub. 3)

Artificial sentence datasets, produced using general segmentation parameters from Sub. 1, are used for deep neural network training, and all the actual sentence samples are used for recognition testing.

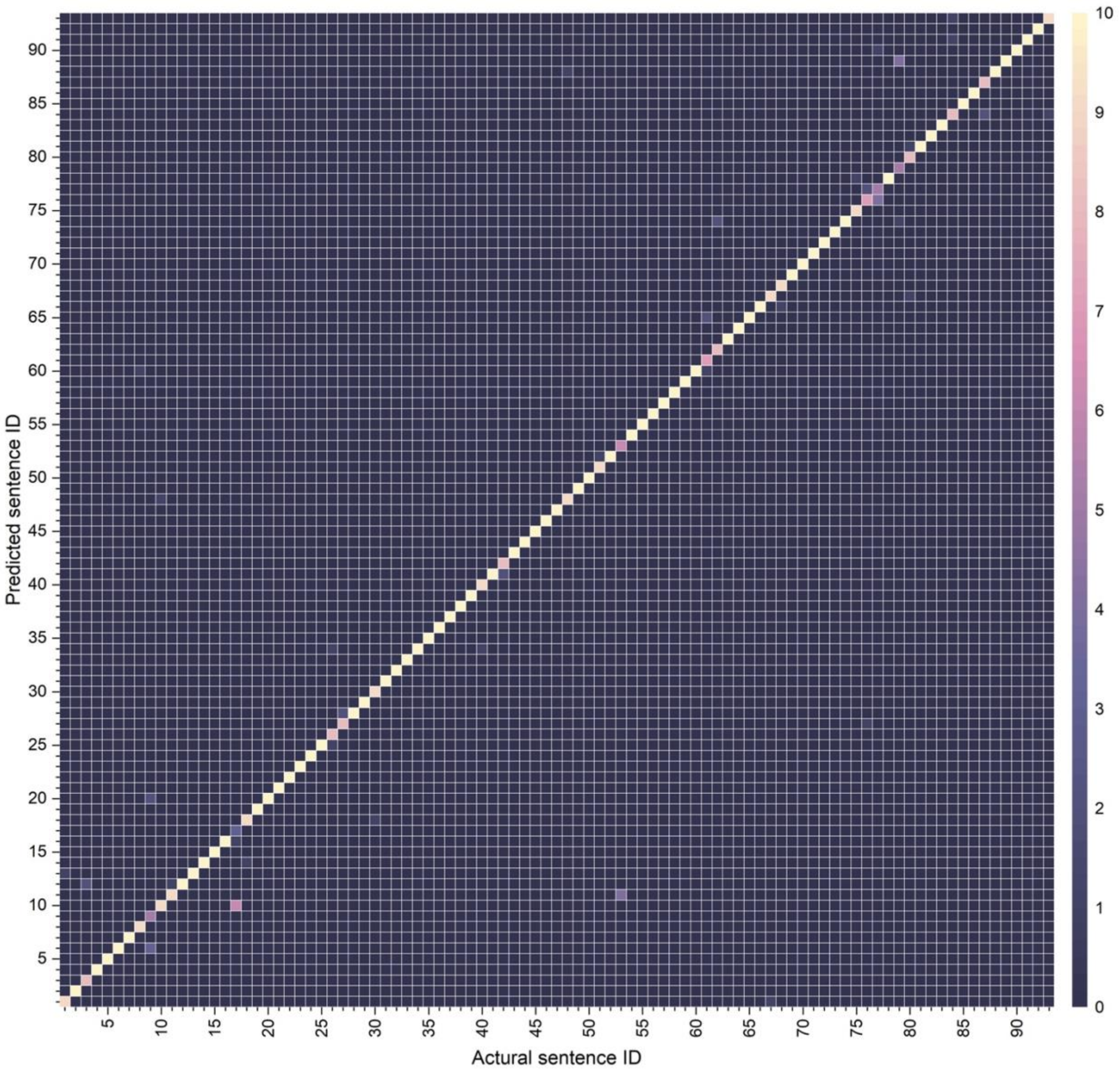

Fig. S18. Recognition accuracy for the 93 sentences, using general parameters (Sub. 4)

Artificial sentence datasets, produced using general segmentation parameters from Sub. 1, are used for deep neural network training, and all the actual sentence samples are used for recognition testing.

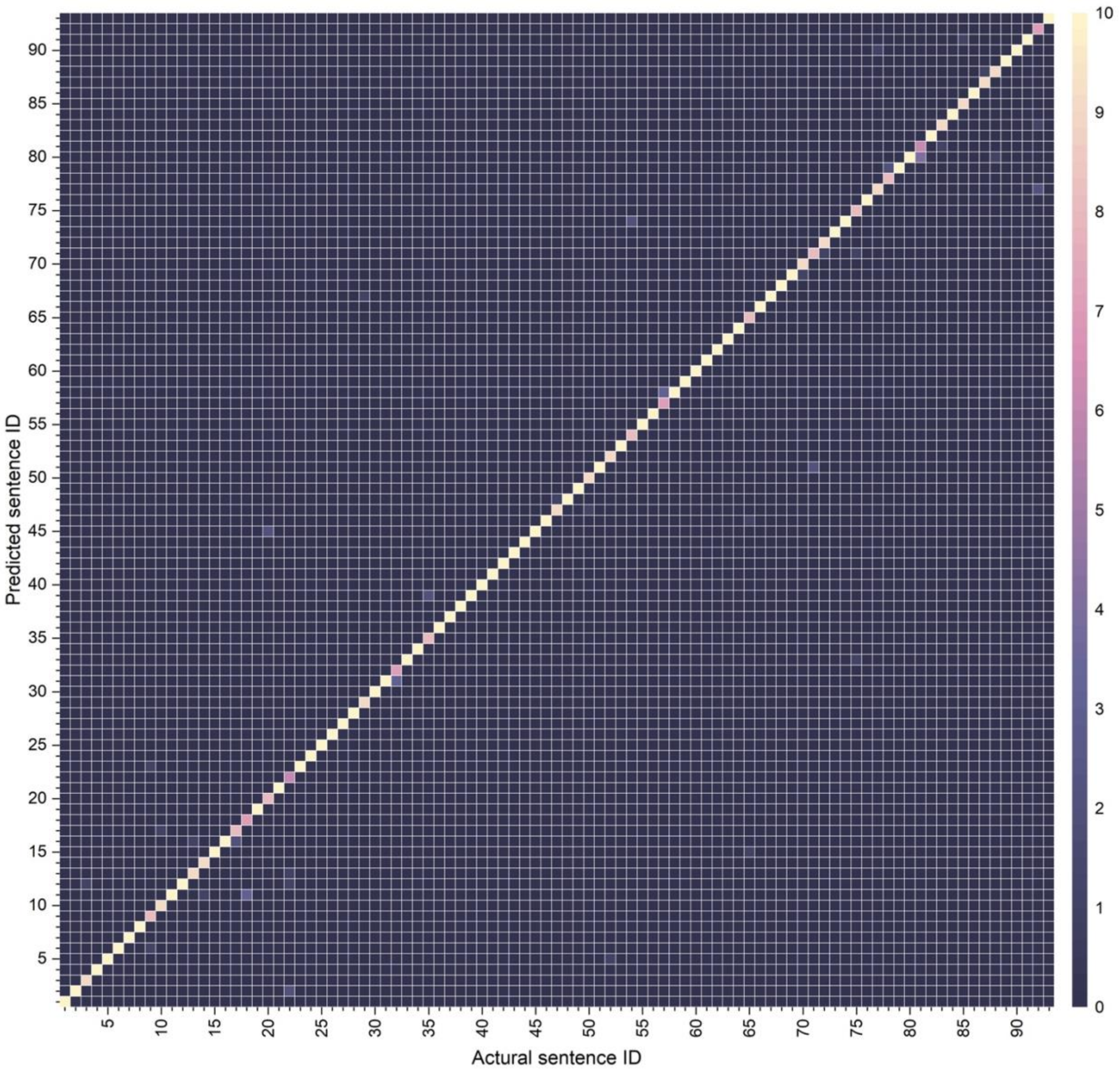

Fig. S19. Recognition accuracy for the 93 sentences, using general parameters (Sub. 5)

Artificial sentence datasets, produced using general segmentation parameters from Sub. 1, are used for deep neural network training, and all the actual sentence samples are used for recognition testing.

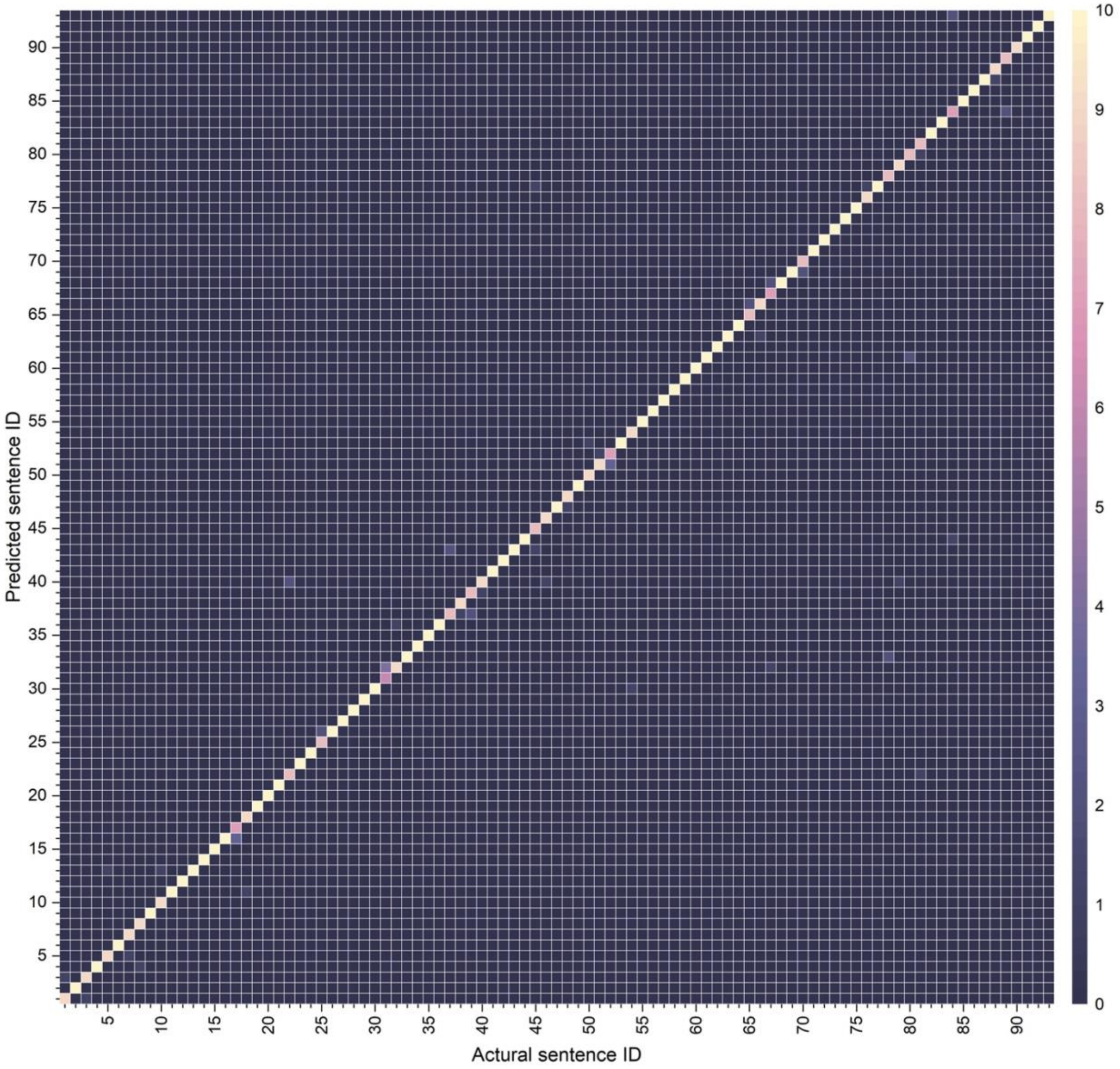

Fig. S20. Recognition accuracy for the 93 sentences, using general parameters (Sub. 6)

Artificial sentence datasets, produced using general segmentation parameters from Sub. 1, are used for deep neural network training, and all the actual sentence samples are used for recognition testing.

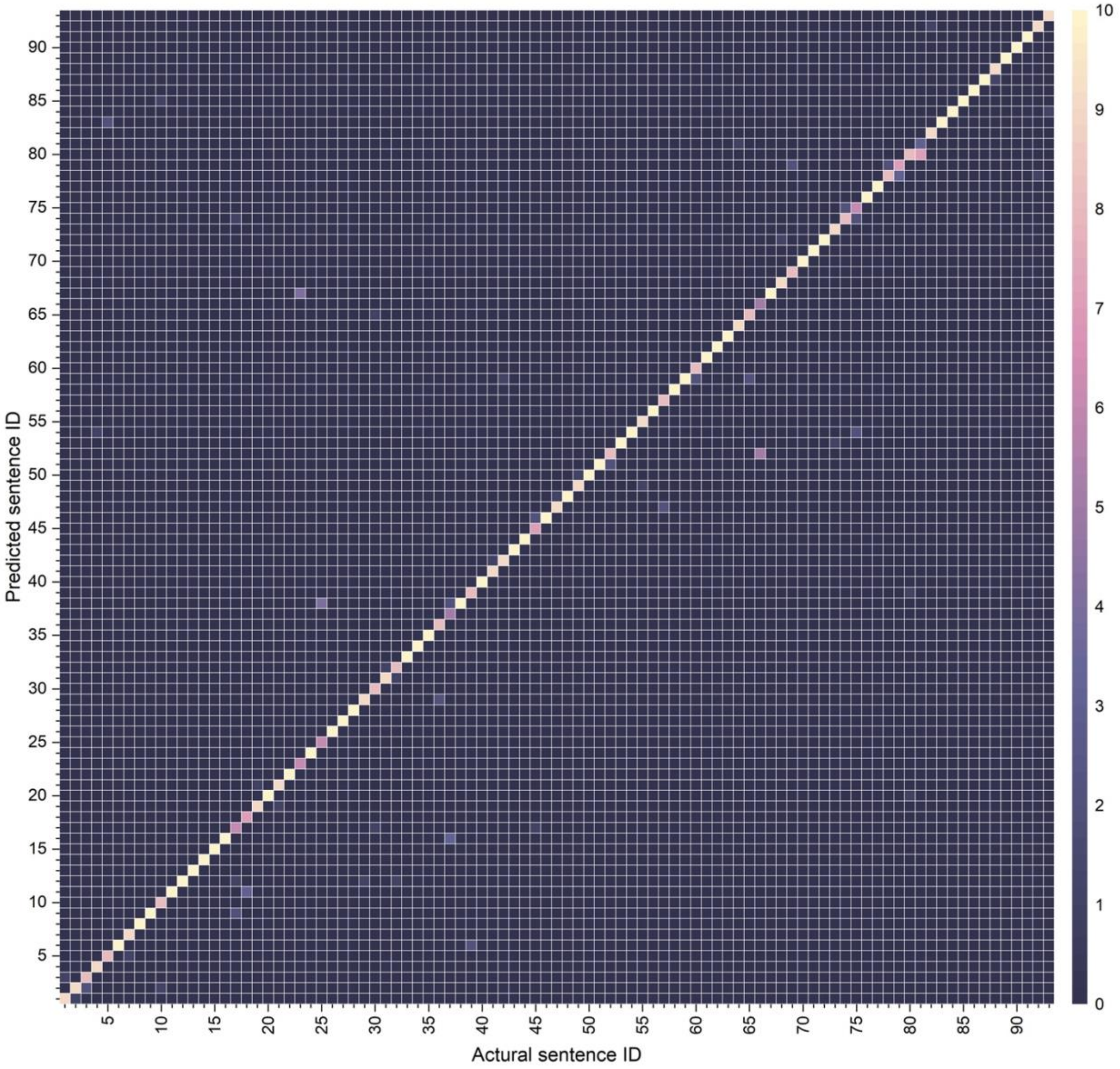

Fig. S21. Recognition accuracy for the 93 sentences, using general parameters (Sub. 7)

Artificial sentence datasets, produced using general segmentation parameters from Sub. 1, are used for deep neural network training, and all the actual sentence samples are used for recognition testing.

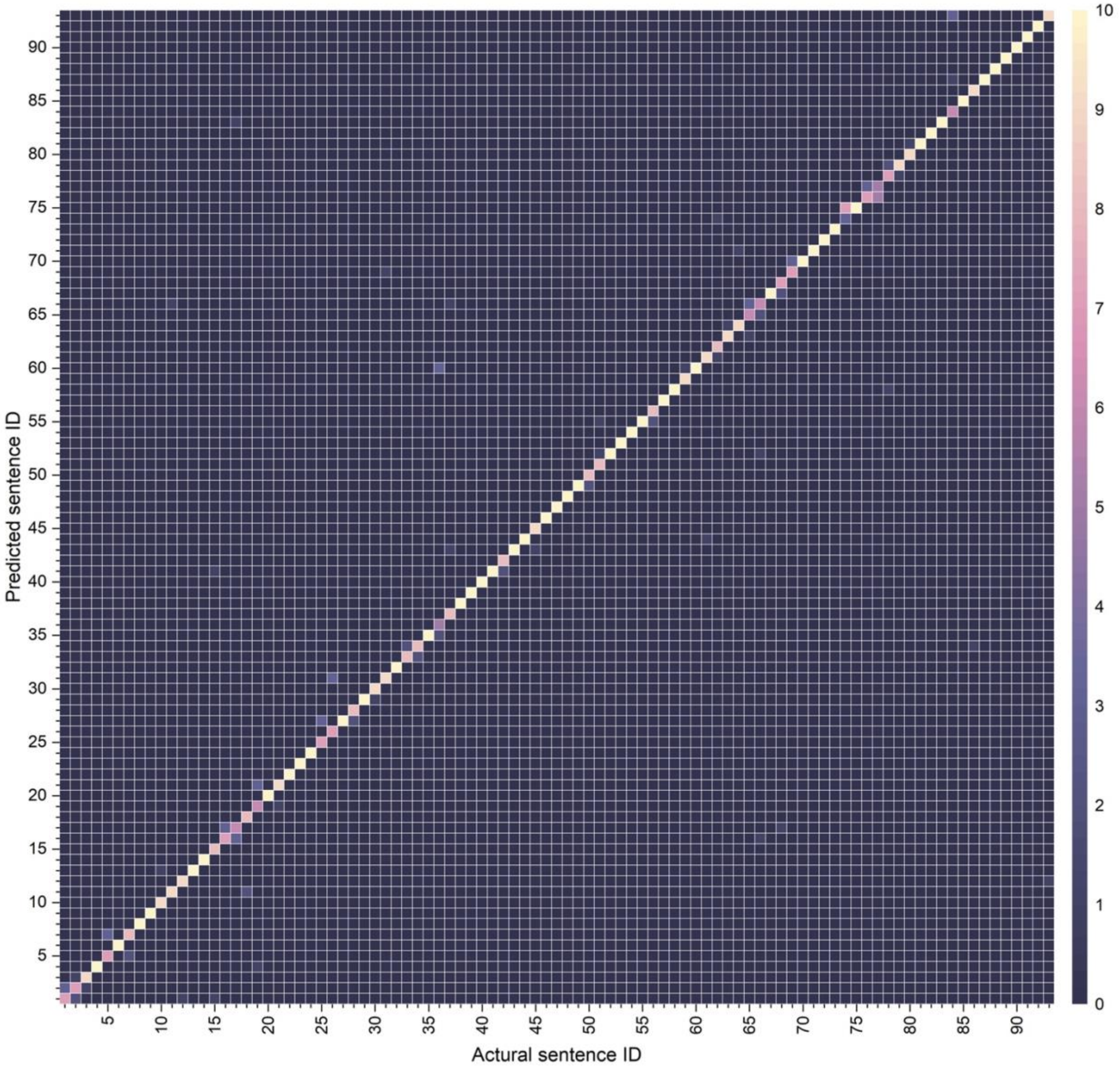

Fig. S22. Recognition accuracy for the 93 sentences, using general parameters (Sub. 8)

Artificial sentence datasets, produced using general segmentation parameters from Sub. 1, are used for deep neural network training, and all the actual sentence samples are used for recognition testing.

No. (ID)	Word (phrase)	No. (ID)	Word (phrase)	No. (ID)	Word (phrase)
1	nice	32	interesting	63	peach
2	glad	33	exciting	64	peanut
3	pleased	34	amazing	65	walnut
4	to	35	annoying	66	olive
5	meet	36	want	67	pomelo
6	you	37	have	68	hello
7	good	38	breakfast	69	smart
8	luck	39	lunch	70	home
9	morning	40	need	71	assistant
10	afternoon	41	some	72	open
11	evening	42	bread	73	close
12	night	43	pizza	74	the
13	see	44	noodles	75	door
14	later	45	dumplings	76	window
15	thank	46	fish	77	turn-on
16	I	47	pasta	78	turn-off
17	am	48	salad	79	light
18	sorry	49	tomato	80	conditioner
19	excuse	50	potato	81	play
20	me	51	water	82	stop
21	yes	52	milk	83	music
22	do	53	juice	84	wheelchair
23	no	54	coffee	85	go
24	don’t	55	like	86	forward
25	feel	56	apple	87	backward
26	happy	57	banana	88	right
27	sad	58	strawberry	89	left
28	sick	59	coconut	90	upstairs
29	fine	60	grape	91	downstairs
30	it	61	mango	92	quickly
31	is	62	orange	93	slowly

Table S1. The 93 English words used for investigation. Each word uses its position in the list as the word ID.

No. (ID)	Sentence	No. (ID)	Sentence	No. (ID)	Sentence
1	Nice to meet you	32	I don’t need fish	63	I like peach
2	Glad to meet you	33	I want some pasta	64	I don’t like peach
3	Pleased to meet you	34	I don’t need pasta	65	I like peanut
4	Good luck	35	I want some salad	66	I don’t like peanut
5	Good morning	36	I don’t need salad	67	I like walnut
6	Good afternoon	37	I want some tomato	68	I don’t like walnut
7	Good evening	38	I don’t need tomato	69	I like olive
8	Good night	39	I want some potato	70	I don’t like olive
9	See you later	40	I don’t need potato	71	I like pomelo
10	Thank you	41	I want some water	72	I don’t like pomelo
11	I am sorry	42	I don’t need water	73	Hello smart home assistant
12	Excuse me	43	I want some milk	74	Open the door
13	Yes, I do	44	I don’t need milk	75	Close the door
14	No, I don’t	45	I want some juice	76	Open the window
15	I feel happy	46	I don’t need juice	77	Close the window
16	I feel sad	47	I want some coffee	78	Turn-on the light
17	I feel sick	48	I don’t need coffee	79	Turn-off the light
18	I am fine	49	I like apple	80	Turn-on the conditioner
19	It is interesting	50	I don’t like apple	81	Turn-off the conditioner
20	It is exciting	51	I like banana	82	Play music
21	It is amazing	52	I don’t like banana	83	Stop music
22	It is annoying	53	I like strawberry	84	Hello smart wheelchair
23	I want some bread	54	I don’t like strawberry	85	Go forward
24	I don’t need bread	55	I like coconut	86	Go backward
25	I want some pizza	56	I don’t like coconut	87	Go right
26	I don’t need pizza	57	I like grape	88	Go left
27	I want some noodles	58	I don’t like grape	89	Go upstairs
28	I don’t need noodles	59	I like mango	90	Go downstairs
29	I want some dumplings	60	I don’t like mango	91	Go quickly
30	I don’t need dumplings	61	I like orange	92	Go slowly
31	I want some fish	62	I don’t like orange	93	Stop smart wheelchair

Table S2. The 93 English sentences used in the investigation. Each sentence uses its position in the list as the sentence ID.

Table S3. The general segmentation parameters for artificial lip speech data generation of all subjects.

Movie S1. Demonstration of our method during sentence speech under different speech speed.

A TCN-based neural network is used to model the decoder for lip language recognition here. Artificial datasets for all 93 sentences including those synthesized using individual optimal segmentation parameters and those generated by data augmentation are used for network training. Vocal speech under different speed (normal and slow) of 12 sentences from the 93-sentence corpus is used for demonstration. Subject 1 participates the video demonstration. The computer displays the recognized sentence on the screen and read it aloud at the same time. The video of the subject and the related signal display are recoded simultaneously. The signals of lip speech displayed on the PC in the video are enlarged and shown behind the video. From top to bottom, the first four signals are the relative attitude angles of chin (θ_{C}, and γ_{C}) and lip (θ_{L} and γ_{L}), and the bottom red line of signal indicates the mouth activities including both speaking and non-speaking (e.g., the minor lip movement induced by swallowing, only about 10 sampling periods). The unit of the horizontal axis here is sampling period/point.

Movie S2. Demonstration of our method during sentence speech while speaking silently.

The same TCN-based neural network model with that in Movie S1 is used for lip language recognition here. Silent speech of the same 12 sentences with those in Movie S1 is used for demonstration. Subject 1 participates the video demonstration too. The computer displays the recognized sentence on the screen and read it aloud at the same time. The video of the subject and the related signal display are recoded simultaneously. The signals of lip speech displayed on the PC in the video are enlarged and shown behind the video. From top to bottom, the first four signals are the relative attitude angles of chin (θ_{C}, and γ_{C}) and lip (θ_{L} and γ_{L}), and the bottom red line of signal indicates the mouth activities including both speaking and non-speaking (e.g., the minor lip movement induced by swallowing, only about 10 sampling periods). The unit of the horizontal axis here is sampling period/point.
